# A forecast of staple crop production in Burkina Faso to enable early warnings of shortages in domestic food availability

**DOI:** 10.1038/s41598-022-05561-9

**Published:** 2022-01-31

**Authors:** Rahel Laudien, Bernhard Schauberger, Jillian Waid, Christoph Gornott

**Affiliations:** 1 Member of the Leibniz Association, Potsdam Institute for Climate Impact Research (PIK), P.O. Box 60 12 03, 14412 Potsdam, Germany; 2 Department of Sustainable Agriculture and Energy Systems, University of Applied Sciences Weihenstephan-Triesdorf, Am Staudengarten 1, 85354 Freising, Germany; 3 Agroecosystem Analysis and Modelling, Faculty of Organic Agricultural Sciences, University of Kassel, Mönchebergstraße 19, 34109 Kassel, Germany

**Keywords:** Climate change, Projection and prediction, Plant sciences, Climate-change adaptation

## Abstract

Almost half of the Burkinabe population is moderately or severely affected by food insecurity. With climate change, domestic food production may become more under pressure, further jeopardizing food security. In this study, we focus on the production of maize, sorghum and millet as staple cereal crops in Burkina Faso to assess food availability as one component of food security. Based on a statistical weather-driven crop model, we provide a within-season forecast of crop production 1 month before the harvest. Hindcast results from 1984 to 2018 produce an r^2^ of 0.95 in case of known harvest areas and an r^2^ of 0.88 when harvest areas are modelled instead. We compare actually supplied calories with those usually consumed from staple crops, allowing us to provide early information on shortages in domestic cereal production on the national level. Despite the—on average—sufficient domestic cereal production from maize, sorghum and millet, a considerable level of food insecurity prevails for large parts of the population. We suggest to consider such forecasts as an early warning signal for shortages in domestic staple crop production and encourage a comprehensive assessment of all dimensions of food security to rapidly develop counteractions for looming food crises.

## Introduction

Agriculture in Burkina Faso is primarily subsistence-based and rainfed. The dependence on favourable weather conditions, such as sufficient and well-distributed rainfall and a reliable onset and length of the rainy season^1^, make agricultural production in Burkina Faso particularly vulnerable to climate change and altered weather variability. Cereal yields are expected to decrease by 18% in the Northern Sudano-Sahelian countries due to the impact of higher temperatures, which lead to a reduced crop cycle duration and increased evaporation rates and thus water stress^2–4^.

From 2017 to 2019, nearly half of the Burkinabe population was moderately or severely affected by food insecurity^5^. Due to ongoing armed conflicts and the outbreak of COVID-19 in 2020, which negatively affected households’ income and access to markets, the number of food insecure people is even expected to increase^6^. This underlines the need to study the reasons and devise tools for increasing or stabilizing food supply.

A within-season yield forecast provides information about the expected harvest. This early warning can allow governments to adjust food imports in case of expected harvest losses^7–10^ and ask for external food assistance to alleviate food shortages. Yield forecasts can therefore support food security planning in face of unfavourable weather conditions.

Whereas existing yield forecasting studies in Burkina Faso focus on single crops, have a limited geographic coverage or time horizon^11–14^, our study covers the whole country and provides a within-season yield forecast for the most important cereal crops of maize, sorghum and millet. In addition to a statistical weather-driven crop yield model tested for the time period from 1984 to 2018, we use information about harvest areas to derive a crop production forecast 1 month before the harvest. We rigorously validate our forecasts in two levels of out-of-sample modelling.

Moreover, we complement existing studies on food security in Burkina Faso—e.g. focusing on food access^15,16^, off-farm income^17,18^ or dietary diversity^19^—by comparing the supplied calories from staple crops with the historic demand on national level. This allows us to provide early information on shortages in domestic cereal production on the national level. Following this approach, our study aims to contribute to a better understanding of food production as one component of food availability in Burkina Faso.

## Results

First, we present models to estimate crop yields for maize, sorghum and millet in Burkina Faso at the end of the growing season, i.e. assuming all relevant weather information is known. Combining crop yields with harvest area estimations, we model crop production as yield times harvest area. Second, the estimation models are turned into forecasting models of cereal-based calories by withholding weather information from the last month of the growing season. The produced calories from maize, sorghum and millet are then compared to the calories usually consumed from those crops.

### Performance in estimating yields, area and production

**Figure 1:**
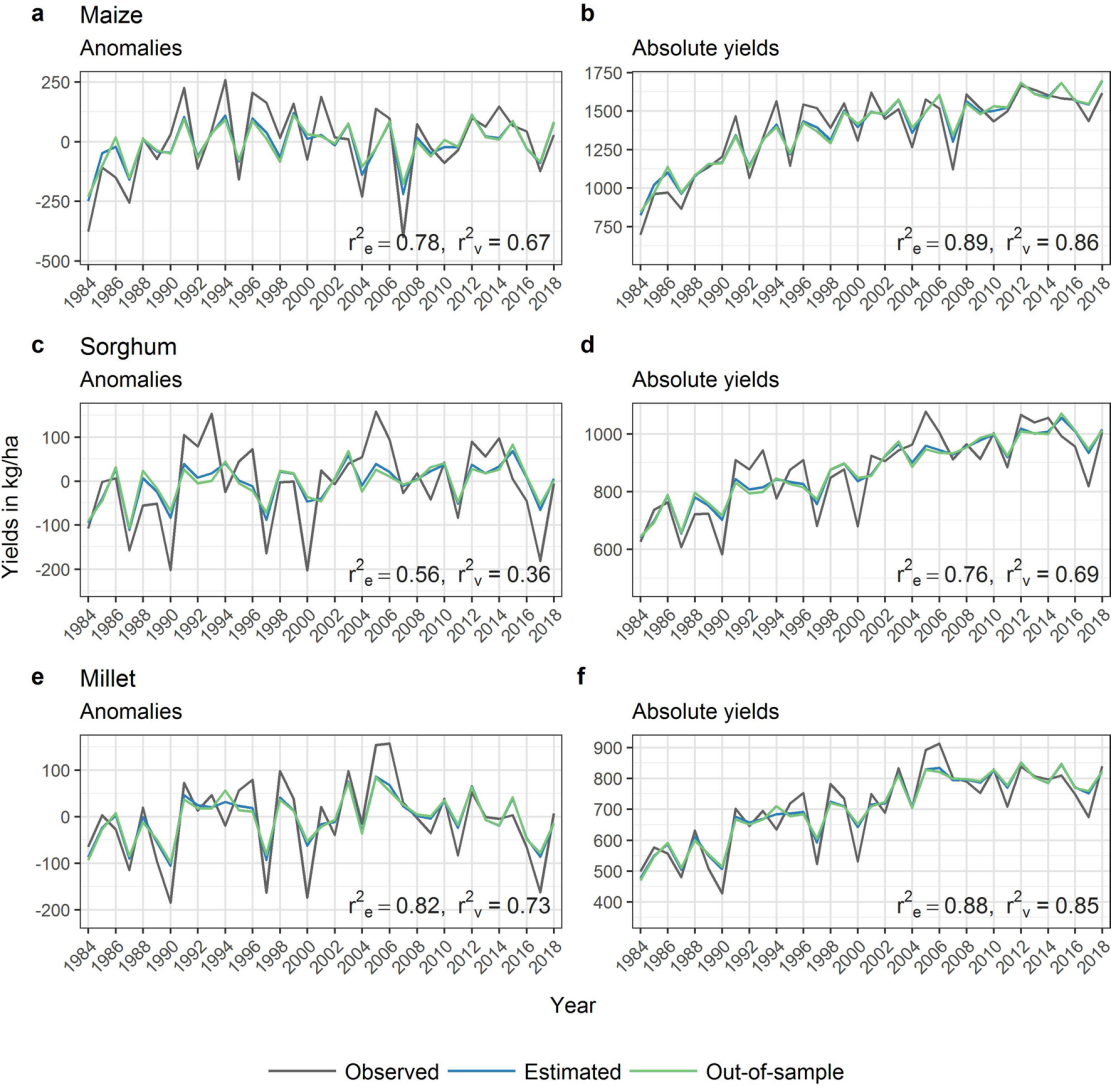
Crop model performance for yield anomalies (left column) and absolute yields (right column) for maize, sorghum and millet from 1984 to 2018. The plot shows the observed yields in grey, the estimation results in blue and the out-of-sample validation results in green. The r^2^_e_ and r^2^_v_ values indicate the explained variance by each model, respectively. The province-specific model performances are shown in SI Fig. 1, a map with province names is shown in SI Fig. 2.

The yield model shows that variation in crop yield anomalies, i.e. fluctuations around an underlying trend, is highly influenced by variations in weather (Fig. 1). This is particularly evident for maize and millet. The out-of-sample results (i.e. the validation based on independent test data) indicate a share of 73% of yield variation for maize and 67% of yield variation for millet, explained by variations in weather. For sorghum, the weather-driven variation in yield is lower with 36%. If absolute yields are considered instead of anomalies, the out-of-sample validated explanatory power of our models increases to 86%, 85% and 69% for maize, millet and sorghum, respectively (Fig. 1). The weather-driven yield models perform better than a constant model that estimates the mean yield excluding the current year (SI Table 1). Together with the high out-of-sample explained variances, this suggests that the weather influences on crop yields are robustly detected, allowing for assessing yield forecasts based on weather observations.

**Figure 2:**
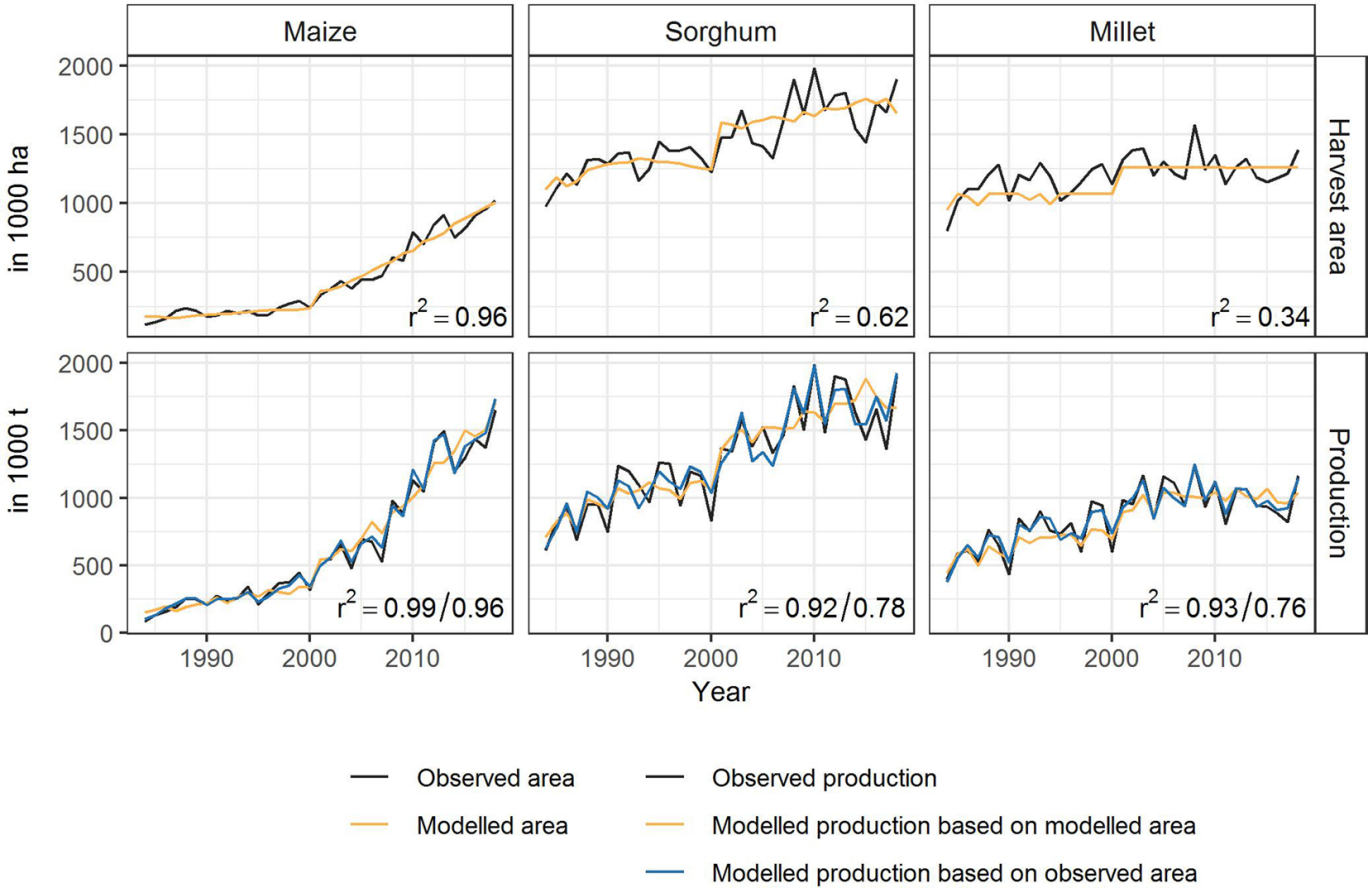
Harvest area and production for maize, sorghum and millet between 1984 and 2018. The black lines show the observed harvest area and production. For area, the best out-of-sample model was chosen. The modelled production is shown for two cases: (1) harvest area is known through e.g. farmer survey or satellite imageries (blue, first r^2^ value); (2) harvest area is unknown and was modelled (yellow, second r^2^ value).

To model crop production, information about harvest areas is required. As this might not be available in practice, we devised empirical models for harvest areas. The best out-of-sample fit to observed harvest areas can be achieved with the LOESS-based trend for maize and sorghum and the median harvest area for millet, resulting in explained variations of 96% for maize, 62% for sorghum and 34% for millet, respectively (Fig. 2, top row).

Combining yield and area estimations, we modelled crop production as yield times harvest area. We compared two versions: one where the harvest area is assumed as known and one where the harvest area is modelled. For yields, we used the model results of the out-of-sample variable selection. In the case of modelled harvest areas, 96% of observed production variability can be explained for maize, 78% for sorghum and 76% for millet. In case of known harvest areas, the explained variability of production increases to 99% for maize, 92% for sorghum and 93% for millet (Fig. 2).

### Hindcast performance of the forecast of supplied calories compared to the usually consumed calories from staple crops

We turned the production models into forecasting models by withholding weather information from the last month of the growing season. Furthermore, production amounts of maize, sorghum and millet were converted into calories and added up to get the calories production of staple crops on national level. This allows us to compare produced calories with the historic demand for calories from staple crops in Burkina Faso.

**Figure 3:**
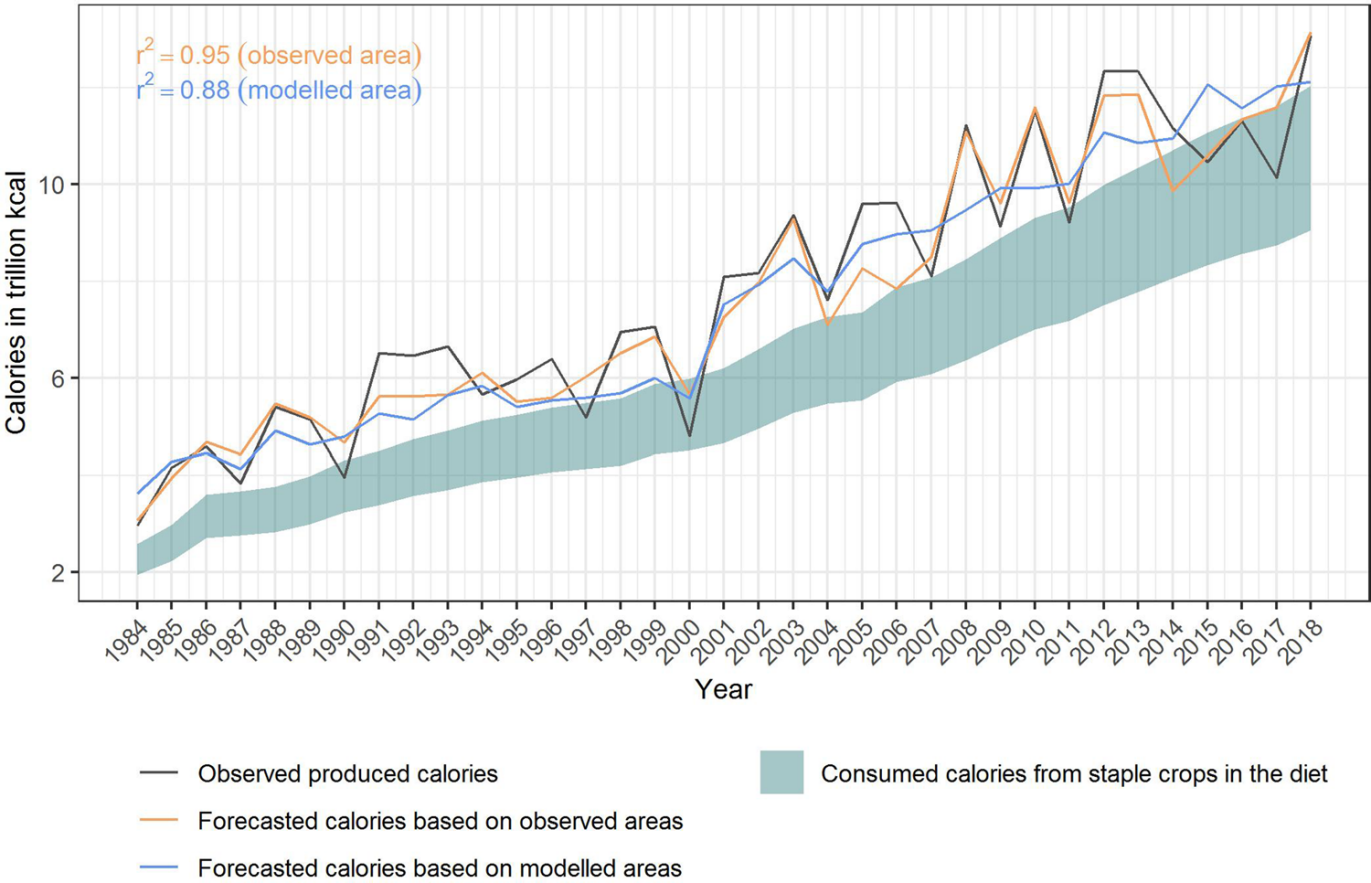
Hindcast performance of aggregated nationally produced calories from staple crops (maize, sorghum and millet) compared to the usually consumed calories from those crops in Burkina Faso. The black line shows the observed produced calories minus post-harvest losses and the bran. The forecast is provided for two cases: (1) harvest areas are known (yellow line), (2) harvest areas are not known and modelled instead (blue line). The band shows the range between the lowest and the highest share of calories from maize, sorghum and millet from 1984 to 2018 in the total supplied calories (i.e. between ca. 47% and 61% of total supplied calories stemmed from maize, sorghum and millet in the past in Burkina Faso).

Assuming unknown, i.e. modelled, harvest areas, the production forecast for maize, sorghum and millet agrees strongly with observed production (r^2^ = 0.88). With known harvest areas, the r^2^ increases to 0.95 and the forecast more accurately represents the amplitude of the peaks (Fig. 3). Notably, this high agreement was achieved with a rigorous validation (level 2: out-of-sample variable selection), where no yield information from the year to be forecasted was used in model construction and estimation—which is similar to an operational context. Crop-specific production forecasts are shown in SI Fig. 3.

From 1984 to 2018 the domestic demand for calories from maize, sorghum and millet has increased in Burkina Faso due to a growing population (SI Fig. 4). Supplied calories from maize, sorghum and millet have increased by enhancing yields and expanding harvest areas (SI Fig. 5). The produced calories (excluding post-harvest losses and the bran) exceeded or fell within the range of usually consumed calories from those crops in the past on nationally aggregated level (Fig. 3). On average, there were 23% more calories produced than consumed.

## Discussion

Our study presents models to estimate staple crop yields (maize, sorghum and millet) and harvest areas in Burkina Faso, and combines these into within-season crop production forecasts. Our rigorously validated production forecasts show very high agreement with actually observed production. Thus, the forecast could support near-term planning endeavours to adjust food trade balances, mobilize disaster reliefs and food aid in case of expected harvest losses by anticipating deficiencies in calories supply.

Weather influences can explain a substantial part of crop yield variations in Burkina Faso as indicated by the high performance of the crop yield model in the out-of-sample validation (r^2^ between 0.73 (0.86) and 0.36 (0.69) for yield anomalies (or absolute yields, respectively)). Our linear regression models provide robust and interpretable results, which is important in an operational context. As the increasing trend in absolute yields acts as a major driver of explanatory power, the model performance is greater for absolute yields compared to yield anomalies. As the yield estimation quality thus relies on a persisting trend, at least for the next year, a causal analysis of the recent increases (management, fertilizer, improved seeds, irrigation expansion etc.) is recommended, but beyond the scope of this study.

The yield forecast (SI Fig. 6) shows similarly good results compared to the yield model, which suggests that the vegetative and first part of the reproductive phase of the growing season sufficiently cover important weather influences. This is in line with findings of Schauberger et al.^20^, who also found a high and sometimes even higher model performance for a reduced growing season, omitting the last part of the reproductive phase. The weather-based yield forecast performed better than a model based on trends only, indicating the relevance of weather for yield formation (SI Fig. 7).

To our best knowledge, there is no previous study covering the same regional scope and time period of crop estimation in Burkina Faso. Yet, our results are partly comparable to two local studies. Our model skill is higher for maize and millet and lower for sorghum compared to the weather-based yield model of Belesova et al.^21^ for the province Kossi and the NDVI-based yield forecast of Karst et al.^14^ for the department Nouna within Kossi. The lower performance for sorghum could be related to reliability issues in the sorghum data applied here (SI Text 1).

To forecast crop production in Burkina Faso, information about crop yields and harvest areas is required, as production is a function of both. For hindcasting past production, the actual area is available. But this is not necessarily the case during the growing season, although early area information would be crucial for early production estimation. While, in principle, farm surveys or satellite observations could detect sown areas during the season, handling frictions and temporal latencies might obfuscate their reliability in practice. Therefore, we estimated this year’s harvest areas based on information from preceding years—assuming persistent trends as for yields. Using this approach, harvest areas can be represented to a large extent for maize and sorghum due to ongoing upward trends in area. Trends in millet areas are less pronounced and thus less indicative for areas in the target year. Calculated crop production shows a high agreement in case of known harvest areas (r^2^ > 0.9) as well as modelled harvest areas (r^2^ > 0.75).

Our study provides the first production forecast covering the whole country and the main staple cereal crops in Burkina Faso. The lead time of the crop-specific forecast (SI Fig. 6) is 1 month for each crop (SI Fig. 8). Due to possible delays in the provision of climate data, different harvest dates of the crops (at the forecasting time of sorghum and millet, maize is already harvested) and possible dissemination issues, the lead time of the aggregated production forecast is lower in practice. Nonetheless, we assume that forecasts for the single crops and also real-time estimates of aggregated available production around harvest time can convey valuable information for policy makers. To provide transparency about the forecasting skill, we use two validations. In addition to a classic (‘jack-knife’) out-of-sample validation, we use a more rigorous out-of-sample variable selection that mimics the operational setting. This validation is rarely used in forecasting^22^ and has also not been applied in existing yield forecasting studies in Burkina Faso^11–14^. Production forecasts for maize, sorghum and millet together, derived under this mock-operational setting, agree strongly with observed production (r^2^ = 0.88 for modelled areas). Thus, we believe that our production forecast could contribute to existing operational food security platforms, such as the Famine Early Warning Systems Network (FEWS NET) which provides food security updates for Burkina Faso incorporating information on crop production, climate, markets, conflicts and nutrition^23^.

In addition to the production forecast, we compare produced and usually consumed calories from staple cereals in the past and assume that a similar share in total food supply will also be needed in the current season. To account for uncertainties in this estimate, we considered a wide range between the highest and the lowest proportion of supplied calories within the last 35 years. Nonetheless, the study methodology should be updated regularly to account for changes in dietary preferences and production choices in Burkina Faso—like e.g. the recent increase in domestic production and consumption of rice.

Our results—both from reported and modelled data—suggest that for most years, there were more produced calories from staple crops than the usual amount of consumed calories from these crops on national level. Despite this surplus in the production of staple crops, there is a prevailing high level of food insecurity in Burkina Faso. According to a FAO-led household survey from 2014 to 2019, 48% of the population (ca. 9 Million people) were moderately or severely food insecure and undernourishment affected about 19% of the population from 2017 to 2019^5^ (SI Fig. 9). The discrepancy between the potentially available and consumed calories could have several causes: First, our assumptions made to derive consumed calories might be too rigorous. Post-harvest losses might be higher in reality. Also apart from food and feed, we did not include other uses of staple crops, such as sorghum beer production. Even though beer production consumes a practically relevant amount of sorghum in Burkina Faso (on average 27% of total supplied calories from sorghum from 2014 to 2018^24^), limited data availability did not allow for a quantification of this impact over the whole time period and was therefore neglected (SI Fig. 10). Second, our national level results do not provide insights about the seasonal, spatial and group-specific distribution of food within the country. Food shortages in specific regions (e.g. the Sahel region in Northern Burkina Faso), times during the year (e.g. the dry season) and/or in specific population groups (e.g. subsistence framers)^6^ are therefore not necessarily reflected in annual and national statistics. Future research should therefore take into account the seasonal and spatial dimension of food availability in Burkina Faso. Third, our study does not account for population-specific demand of food. In particular, the rural population and subsistence farmers with labour intense lifestyles^25^ are characterised by a greater dependence on calorie-rich foods such as cereals, tubers and coarse grains and have fewer capacities to diversify the composition of their diets^25^. Therefore, the share of consumed calories from staple crops is likely higher for those vulnerable groups. Our results should therefore be interpreted as representing a lower bound estimate of the actual required calories from staple crops. Fourth, our study focuses on production from staple crops, which is one important component of food availability and thus food security. Deficiencies in the other three components, namely food access, utilization and stability^26^, contribute to the high level of food insecurity even when, on paper, there are more produced calories than consumed. Local increases in food prices, political instability, ongoing conflicts as well as terrorist threats and attacks in Burkina Faso negatively impact food security in Burkina Faso^6^.

In the past, the increase in production was in line with the strong population growth in Burkina Faso (SI Fig. 4). This increase was achieved by enhanced yields and simultaneously expanded agricultural land (SI Fig. 5). The implementation of improved soil and water conservations practices in the first half of the 1980s, and the shift to early maturing varieties led to yield increases^27^. At the same time, the share of arable land increased from ca. 30% in the beginning of the 1980s to ca. 50% in 2018^28^ (SI Fig. 11). Apart from the negative environmental consequences related to this land use change^27^, the increase in arable land is limited because of finite land resources and has already been stagnating for the last 10 years (SI Fig. 11). Also increasing crop yields through agricultural intensification is constrained by limited access to agricultural inputs (improved seeds, fertilizer, plant protection and machinery), environmental boundaries^29^ and challenges in adopting good agricultural practices^30,31^. Both components—yields and acreage—taken together suggest that domestically produced calories might not be sufficient to meet the increasing demand of a growing population in the long term^32,33^. This looming future deficiency may become more pronounced when adverse effects of climate change materialize in crop production. At this point, other dimensions of food security, namely food access, utilization and stability, will become crucial to guarantee sufficient food supply.

## Methods

**Figure 4:**
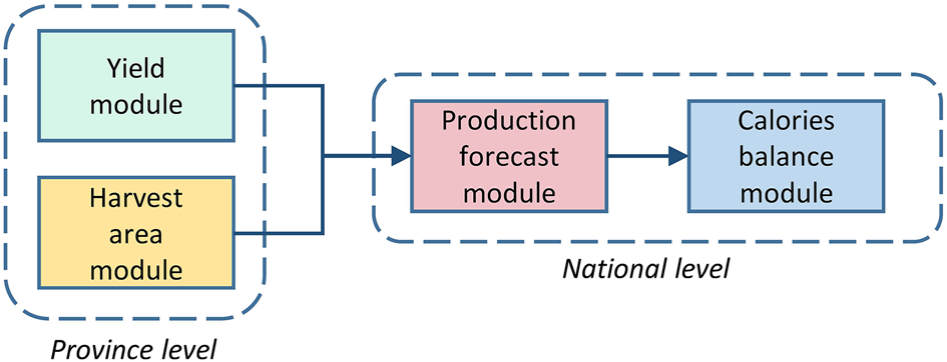
Work flow.

To forecast crop production in Burkina Faso, information about crop yields (harvested amount per area) and harvest areas is required, as production is a function of both. In the following, we describe a yield model (*yield module*) and a harvest area model (*harvest area module*) on province level to calculate both types of information. Both models can be run in an ex-post (estimation of production after the harvest) and an ex-ante (estimation of production before the harvest) mode. In the forecast (ex-ante) mode, the yield model was only supplied with reduced climate information, omitting weather influences of the last month before the harvest. Then, the results of the yield and harvest area model were aggregated to the national level (*production forecast module*). Finally, we compared the produced calories from maize, sorghum and millet with the usually consumed calories from those crops (*calories balance module*; Fig. 4).

For our analysis, we used the statistical software *R*—version 4.0.5^34^. A list of used packages can be found in SI Text 2.

### Yield module

**Figure 5:**
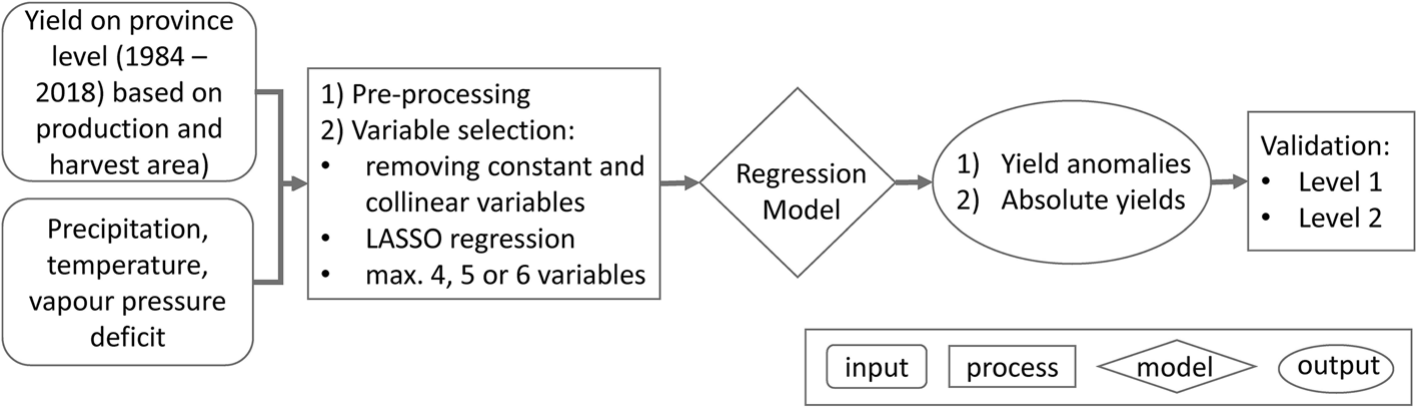
Yield module flow chart.

For each of the 45 provinces, we applied the work flow as shown in Fig. 5, i.e. we set up a separate yield model for each crop and each province. The empirical model comprises crop and province-specific exogenous variables to account for the different growth requirements of each crop type and the diverse climatic conditions within the country.

#### Input data

We used annual harvest area and production statistics for maize, sorghum and millet on province level from 1984 to 2018 from the Burkinabe Ministère de l’Agriculture et des Aménagements Hydroagricoles/Direction Générale des Etudes et des Statistiques Sectorielles^35^. Maize, sorghum and millet are the main cereal crops in Burkina Faso and accounted for 53% of total supplied calories from 1984 to 2016 (median value; SI Fig. 12). Climate data from 1984 to 2018 were extracted from two sources. For precipitation, we used observed daily rainfall totals from CHIRPS (Climate Hazards Group Infrared Precipitation with Stations) at a resolution of 0.25 × 0.25 degrees^36^, which provides reliable precipitation information^37^. For daily mean, maximum and minimum temperature, we used ERA5 data^38^ provided at a resolution of 0.25 × 0.25 degrees. ERA5 is a recent re-analysis product and outperforms ERA-Interim over Africa^39^. Based on the ERA5 temperature data, we calculated vapour pressure deficit^40^ (VPD, SI Eq. 1) to account for water stress caused by high atmospheric water demand, which leads to a reduction in plant carbon uptake^41^ and thus yields. All weather data were mapped to province boundaries, considering only intersecting cells for each province.

#### Exogenous variables

Based on precipitation, temperature and vapour pressure deficit, we created variables that represent yield influencing climate drivers. We included variables that represent the median state of the weather during the growing season, as well as variables accounting for variation and extremes in weather. For precipitation, we took into account the occurrence of consecutive dry and wet days, respectively, and different thresholds for light and heavy precipitation events. SI Table 2 shows a list of all possible model inputs and SI Text 3 further expands on the reasons for the included variables.

The weather variables were calculated for the growing season in Burkina Faso following the FAO crop calendar^42^. All sowing and harvesting activities happen between May and November. As the yield statistics do not provide variety-specific information, we used the median sowing and harvest dates of all reported varieties (SI Fig. 13 for maize and SI Fig. 14 for sorghum and millet). For sorghum and millet the same crop calendar was used because the FAO does not provide a specific calendar for sorghum and due to the similarity in sowing and harvest dates^6^.

The variables were separately calculated for the vegetative (veg), the first (repro1) and the last part of the reproductive phase (repro2) of the growing season. The days in the growing season until 50% of the full-season growing degree days sum (GDD, SI Eq. 2) was reached were allocated to the vegetative phase and the remainder to the reproductive phase, following Schauberger et al.^20^. The reproductive phase was then split into two separate phases (repro1 and repro2) with repro2 covering the last month before the harvest and repro1 covering the time between veg and repro2. The separation between repro1 and repro2 accounts for different weather influences on crop growth during the reproductive phase (grain filling and maturity phase)^43^ and enables a forecast with a lead time of 1 month if weather information from repro2 is withheld from model construction.

#### Pre-processing

Due to unreasonable values in the yield data, we applied data cleaning steps detailed in SI Text 1. Moreover, possible trends in the yield time series were removed on province level, by choosing the polynomial trend (mean, linear or quadratic) with the lowest Akaike Information Criterion (AIC). The weather variables were standardized to a mean of 0 and a standard deviation of 1 to allow for a better comparability of the beta coefficients^44^.

#### Variable selection

We applied the following variable selection process to elucidate important variables for explaining yield variability in the different provinces:

- We removed variables that do not show year-to-year variations (i.e. zero variance).
- To avoid multicollinearity, only those variables were selected that are not strongly collinear (i.e. Pearson’s r > 0.7) with another explanatory variable. If a pair of variables was strongly collinear, then the variable with the higher correlation with yield anomalies was retained and the other removed.
- Input selection was done using LASSO regression. Through regularization, LASSO performs a co-variate selection, which improves both the prediction accuracy and the interpretability^45^. To select the optimum lambda, i.e. the regularization penalty for the LASSO regression, we used the lowest cross-validation (years were omitted subsequently) mean squared error (MSE).
- As a last step, we restricted the maximum number of variables to be included in the model to avoid overfitting. Per crop, we tested a maximum of four, five and six variables and selected the set of variables that showed the highest correlation with yields in the out-of-sample validation.

#### Regression model

For each province, we applied a separate regression model (Eq. 1) following the approach of Gornott and Wechsung^46^ and Schauberger et al.^20^.

1 $${y}_{it}= \sum_{k=1}^{K}{\beta }_{ki} {x}_{kit} + {\varepsilon }_{it }$$

with $\beta$ as parameters, $y$ as the demeaned and detrended response variable, $x$ as the standardized explanatory input variable, and $\varepsilon$ as error term, for $K$ variables ($k=1,\dots ,K$), $N$ spatial units ($i=1,\dots ,N$) and $T$ years ($t=1,\dots ,T$). Exogenous variables ${\varvec{x}}$ are specific to each province and crop; all candidate variables are listed in SI Table 1.

In case of autocorrelation and heteroscedasticity of model residuals, which we tested based on the Breusch–Godfrey and the Breusch–Pagan test respectively, we used robust standard errors^47^.

#### Validation

To test the adequacy of our empirical models, we applied two validations:

- Out-of-sample validation: We selected the variables ($x$ in Eq. 1) with the three steps described above based on all observations. Then we subsequently removed observations for 1 year and used the remaining observations to fit the model coefficients for all selected variables ($\beta$ in Eq. 1) and predict yield anomalies for the removed year.
- Out-of-sample variable selection: We subsequently removed observations for 1 year, then selected variables as described above and estimated the model coefficients based on the remaining data. We used this model to predict yield changes for the removed year. This guarantees that no information from the removed year is used for the variable selection or the model estimation. This validation simulates the operational forecasting context, where no yield information from the year to be forecasted is available for model building.

The goodness of fit between the observed yields and the predicted yields was evaluated based on r^2^, which represents the share of explained variance.

#### Yield forecasting

For the within-season forecast of crop yields, we applied the same pipeline of variable selection, model estimation and model validation as described above, but omitted weather information from the last month before harvest (repro2). The resulting lead time is thus 1 month before the harvest (SI Fig. 8).

### Harvest area module

In our study, we compared two types of data availability for harvest areas: fully known and estimated. Data about the harvest areas could be obtained within the season from farmer surveys or satellite imageries. Yet even though this is possible in principle, it might not be available in practice. To still be able to provide a production forecast in an operational context, we tested different options to represent harvest areas based on information from previous years. We tested the following four options: the median harvest area, the median harvest area of the previous 3 (5) years and the trend of the harvest area calculated by a non-parametric LOESS function with a span of 0.9. We chose the option for calculated harvest area that resulted in the highest correlation (Pearson’s *r*) between the observed and the modelled area.

### Production forecast module

Production is calculated as yield times harvest area for each crop and province. To obtain total national production of staple crops, province-level production data for maize, sorghum and millet were added up. In the forecasting case, the lead time of the production forecast of all crops together is 1 month before the sorghum and millet harvest (which happens around end of October/begin of November). The maize harvest (end of September/October) has already happened by then; the implications of this are discussed below.

### Calories balance module

In the calories balance module, we compare the produced calories with the usually consumed calories from staple crops in Burkina Faso. First, production amounts of maize, sorghum and millet were converted into calories and added up to get the calories production of staple crops on national level, using nutrient information^48^ shown in Table 1. Second, we subtracted the share of the bran^48^ (Table 1) from the produced calories, which is used for feed in Burkina Faso^24^. Due to post-harvest losses (PHL), not all produced calories are available for food energy uptake. In the last step we therefore subtracted the PHL fraction from the produced calories. We considered the median PHL per crop (Table 1), as annual information about PHL was not sufficiently available. Information about PHL were obtained from Aphlis^49^ who account for losses incurring during harvesting, drying, handling operations, household and market level storage and transport.

**Table 1:** Calories^48^, median post-harvest losses per crop^49^ and share of bran^48^.

	Maize	Sorghum	Millet
Calories per 100 g in kcal	360	343	340
Median post-harvest loss in %	17	13	11
Bran in %	6	13	14

**Figure 6:**
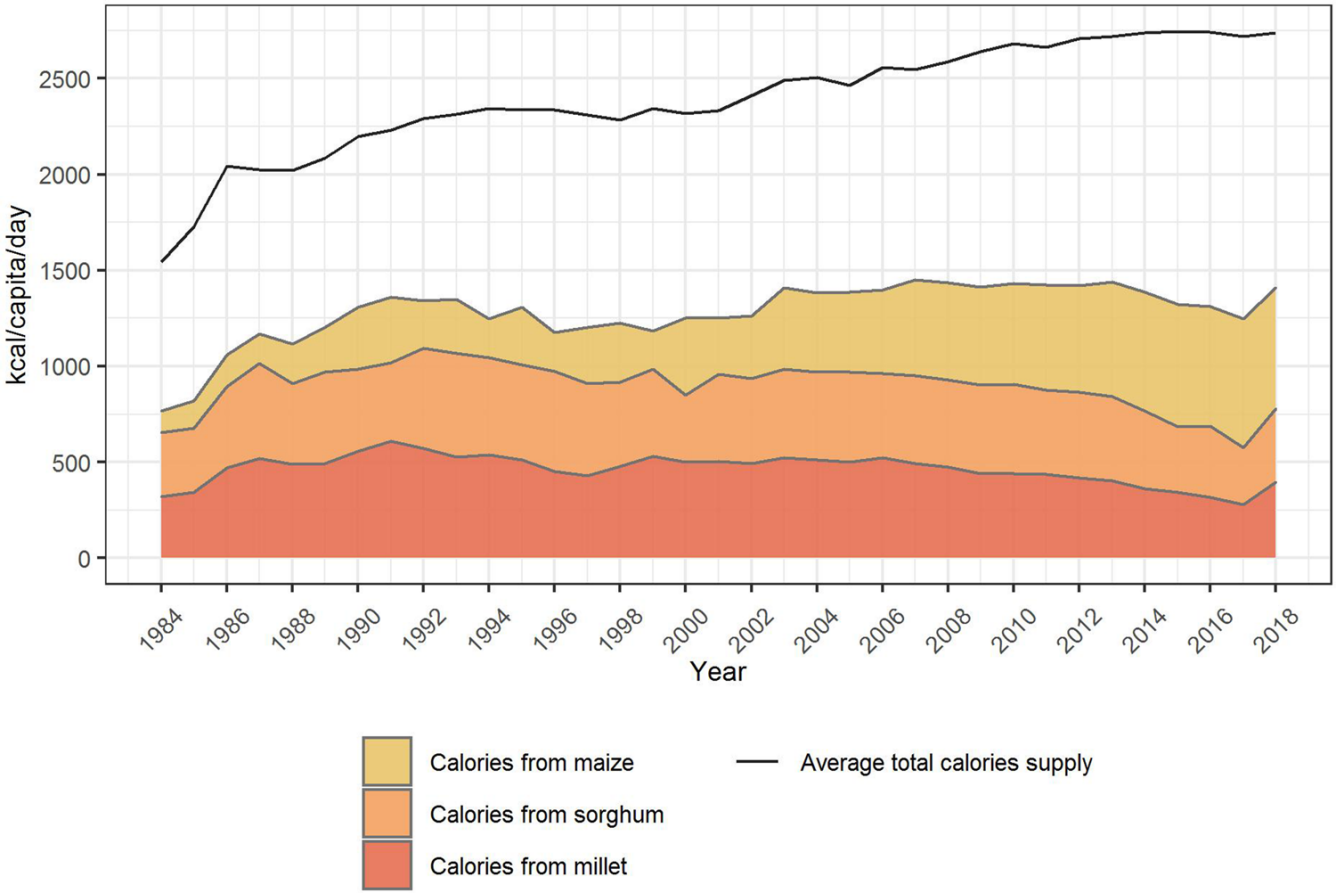
Supplied calories from maize, sorghum and millet and total supplied calories per capita and day in Burkina Faso from 1984 to 2018 in Burkina Faso. The calories supply refers to the total amount of calories available for human food including any commodity derived therefrom as a result of further processing^51^. (source: authors’ illustration based on FAO^51^ and Roser and Ritchie^52^).

The total national consumed calories from staple crops in Burkina Faso were obtained by multiplying the calories from staple crops in the diet per day (Fig. 6) with the number of days per year and the total population in Burkina Faso^50^ (SI Fig. 4). To account for uncertainties in this estimate, we consider the range between the minimum and the maximum share of calories from maize, sorghum and millet in total supplied calories in the time period from 1984 to 2018 and assume that all seasons fall within this long-term average range.

We only took into account domestically produced calories, deliberately disregarding imports and exports. Given the very low amount of cross-border trade for maize, sorghum and millet in Burkina Faso^5^ (SI Fig. 15), we consider domestic production as a good proxy for overall availability.

## Supplementary Information

Supplementary Information.
