# Supplementary material for: A forecast of staple crop production in Burkina Faso to enable early warnings of shortages in domestic food availability
Source: Sci Rep. 2022 Jan 31;12:1638. doi: 10.1038/s41598-022-05561-9 (PMC8803996; doi:10.1038/s41598-022-05561-9)
Supplement: Supplementary file 1 — Supplementary Information. [file 41598_2022_5561_MOESM1_ESM.docx]

Supplementary Information to: “A forecast of staple crop production in Burkina Faso to enable early warnings of shortages in domestic food availability“

**Author names and affiliations:**

Rahel Laudien^a*^, Bernhard Schauberger^b,a^, Jillian Waid^a^, Christoph Gornott^a,c^

^a^Potsdam Institute for Climate Impact Research (PIK)

Member of the Leibniz Association
P.O. Box 60 12 03
D-14412 Potsdam
Germany

^b^University of Applied Sciences Weihenstephan-Triesdorf

Department of Sustainable Agriculture and Energy Systems

Am Staudengarten 1

85354 Freising

Germany

^c^Agroecosystem Analysis and Modelling

Faculty of Organic Agricultural Sciences

University of Kassel

Mönchebergstraße 19

34109 Kassel

Germany

**Corresponding author:**

Rahel Laudien ([laudien@pik-potsdam.de](mailto:laudien@pik-potsdam.de), +49 331 28820771)

**Co-authors:**

Christoph Gornott ([gornott@pik-potsdam.de](mailto:gornott@pik-potsdam.de))

Bernhard Schauberger ([schauber@pik-potsdam.de](mailto:schauber@pik-potsdam.de))

Jillian Waid ([waid@pik-potsdam.de](mailto:waid@pik-potsdam.de))

# Performance of yield models compared to a constant model

|  | Maize | | Sorghum | | Millet | |
| --- | --- | --- | --- | --- | --- | --- |
| Province | **Yield model** | **Constant model** | **Yield model** | **Constant model** | **Yield model** | **Constant model** |
| Bale | 127.97 | 359.31 | 74.66 | 174.24 | 153.19 | 232.24 |
| Bam | 201.48 | 315.59 | 126.78 | 213.99 | 123.73 | 196.71 |
| Banwa | 190.26 | 295.82 | 56.34 | 141.21 | 83.06 | 132.99 |
| Bazega | 158.17 | 251.70 | 121.19 | 161.23 | 110.71 | 137.42 |
| Bougouriba | 243.87 | 319.45 | 121.49 | 222.79 | 122.62 | 170.34 |
| Boulgou | 236.38 | 286.06 | 132.69 | 161.13 | 124.11 | 203.01 |
| Boulkiemde | 298.34 | 366.01 | 153.80 | 186.75 | 131.85 | 159.91 |
| Comoe | 217.64 | 268.60 | 117.48 | 208.43 | 97.42 | 214.23 |
| Ganzourgou | 193.85 | 339.13 | 115.47 | 160.56 | 113.34 | 167.24 |
| Gnagna | 219.55 | 349.28 | 165.70 | 212.43 | 243.78 | 275.28 |
| Gourma | 234.94 | 276.49 | 113.69 | 183.51 | 117.17 | 155.35 |
| Houet | 149.17 | 240.62 | 141.06 | 162.38 | 145.09 | 200.10 |
| Ioba | 188.84 | 240.63 | 86.05 | 166.06 | 95.00 | 151.87 |
| Kadiogo | 303.58 | 403.30 | 161.50 | 231.25 | 156.45 | 181.16 |
| Kenedougou | 116.43 | 225.14 | 92.48 | 147.00 | 158.15 | 198.41 |
| Komandjoari | 177.17 | 437.35 | 127.08 | 203.28 | 32.56 | 224.79 |
| Kompienga | 140.48 | 343.87 | 94.98 | 146.83 | 112.75 | 199.01 |
| Kossi | 192.16 | 295.39 | 131.41 | 158.03 | 76.29 | 129.88 |
| Koulpelgo | 89.02 | 184.27 | 106.10 | 145.33 | 93.61 | 183.21 |
| Kouritenga | 170.04 | 225.56 | 175.61 | 208.54 | 128.11 | 167.18 |
| Kourweogo | 110.92 | 220.49 | 119.95 | 209.40 | 121.01 | 182.00 |
| Leraba | 107.20 | 250.38 | NA | NA | NA | NA |
| Loroum | 130.65 | 389.53 | 146.04 | 227.60 | 80.72 | 238.75 |
| Mouhoun | 195.21 | 339.82 | 104.08 | 126.15 | 78.39 | 135.08 |
| Nahouri | 278.77 | 338.49 | 98.59 | 149.11 | 117.29 | 174.91 |
| Namentenga | 249.75 | 339.83 | 131.18 | 168.69 | 175.48 | 208.40 |
| Nayala | 152.35 | 284.35 | 104.72 | 176.58 | 108.63 | 168.83 |
| Noumbiel | 238.00 | 443.15 | 134.96 | 240.36 | NA | NA |
| Oubritenga | 174.92 | 291.38 | 128.56 | 182.95 | 67.17 | 130.19 |
| Oudalan | 236.91 | 490.12 | 227.39 | 388.39 | 120.37 | 186.64 |
| Passore | 187.42 | 314.66 | 169.86 | 213.45 | 133.60 | 209.52 |
| Poni | 304.77 | 395.60 | 136.84 | 197.02 | 96.88 | 134.01 |
| Sanguie | 333.74 | 430.06 | 96.64 | 151.74 | 98.68 | 141.96 |
| Sanmatenga | 231.67 | 345.30 | 164.87 | 208.87 | 154.42 | 192.04 |
| Seno | 163.36 | 334.26 | 173.51 | 230.12 | 119.20 | 199.39 |
| Sissili | 181.86 | 265.77 | 135.73 | 165.17 | 94.77 | 166.20 |
| Soum | 265.24 | 475.06 | 124.53 | 208.33 | 106.24 | 157.73 |
| Sourou | 355.13 | 493.75 | 187.56 | 218.80 | 158.66 | 190.15 |
| Tapoa | 304.95 | 364.85 | 122.79 | 191.75 | 147.48 | 224.41 |
| Tuy | 66.38 | 298.64 | 64.28 | 120.53 | 117.14 | 192.73 |
| Yagha | 107.47 | 322.41 | 135.44 | 171.12 | 106.58 | 206.18 |
| Yatenga | 187.50 | 322.23 | 145.11 | 166.76 | 141.64 | 205.80 |
| Ziro | 170.50 | 259.33 | 77.63 | 144.45 | 99.06 | 194.60 |
| Zondoma | 83.19 | 330.35 | 81.98 | 194.64 | 113.03 | 248.06 |
| Zoundweogo | 196.85 | 242.16 | 148.10 | 181.03 | 113.29 | 151.57 |

SI Table 1. Comparison of the model performance of yield anomalies to a constant model that only takes the mean yield excluding the year that is forecasted as a predictor for maize, sorghum and millet. The values show the root mean squared error (RMSE) in kg/ha between the observed yield and the modelled yield. A map with province names is provided in SI Fig. 2.

# Province-specific performance of the yield model


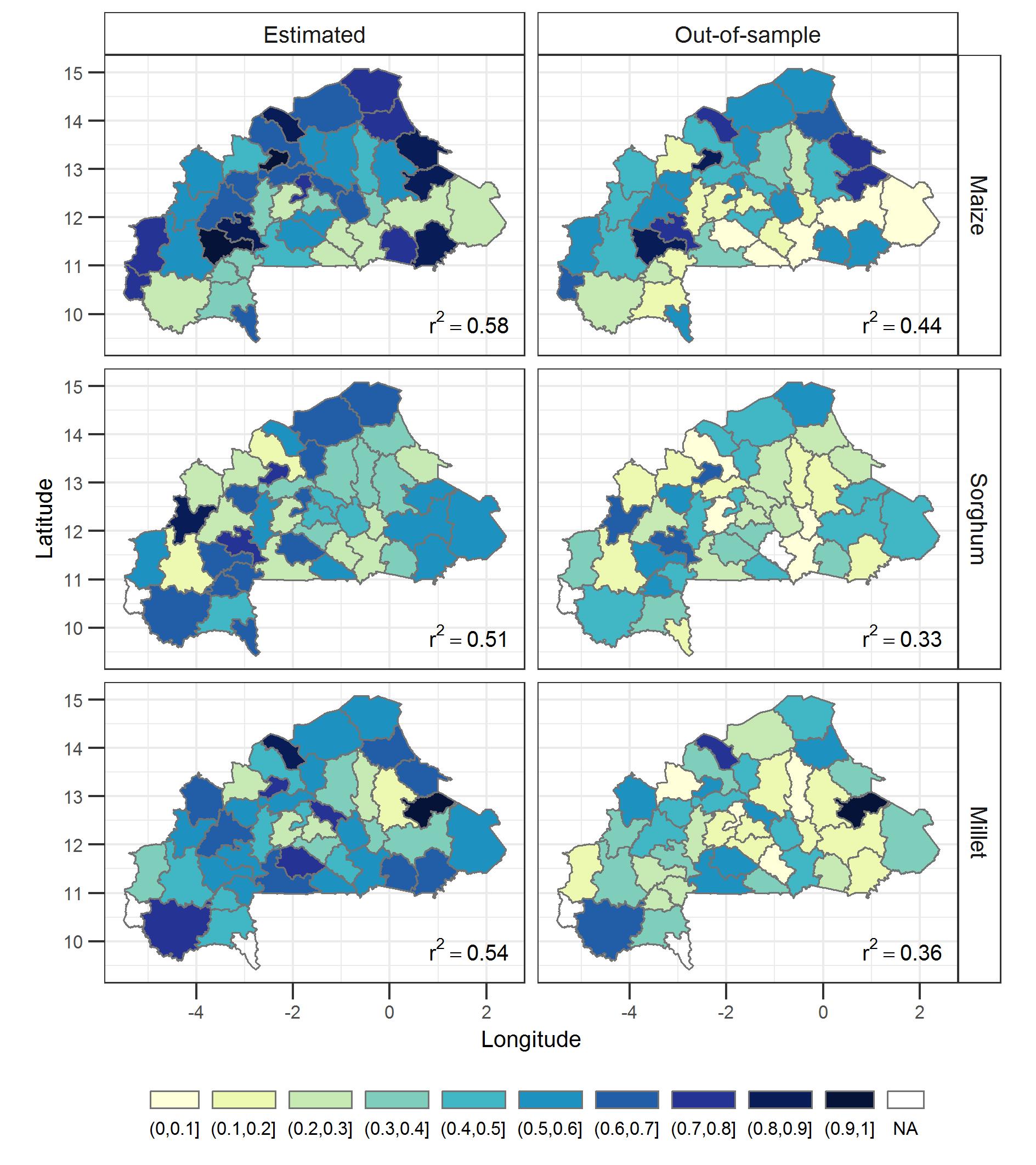


SI Fig. 1. Province-specific performance of the crop model for yield anomalies from 1984 to 2018 measured in r^2^. The left panels show the estimation results (i.e. the model performance when the complete time series for each province is included). The right hand panels show the performance for the out-of-sample validation. The median r^2^ of all provinces in Burkina Faso is shown in the left corner of the panels. A map with province names is provided in SI Fig. 2.

# Map of province names in Burkina Faso


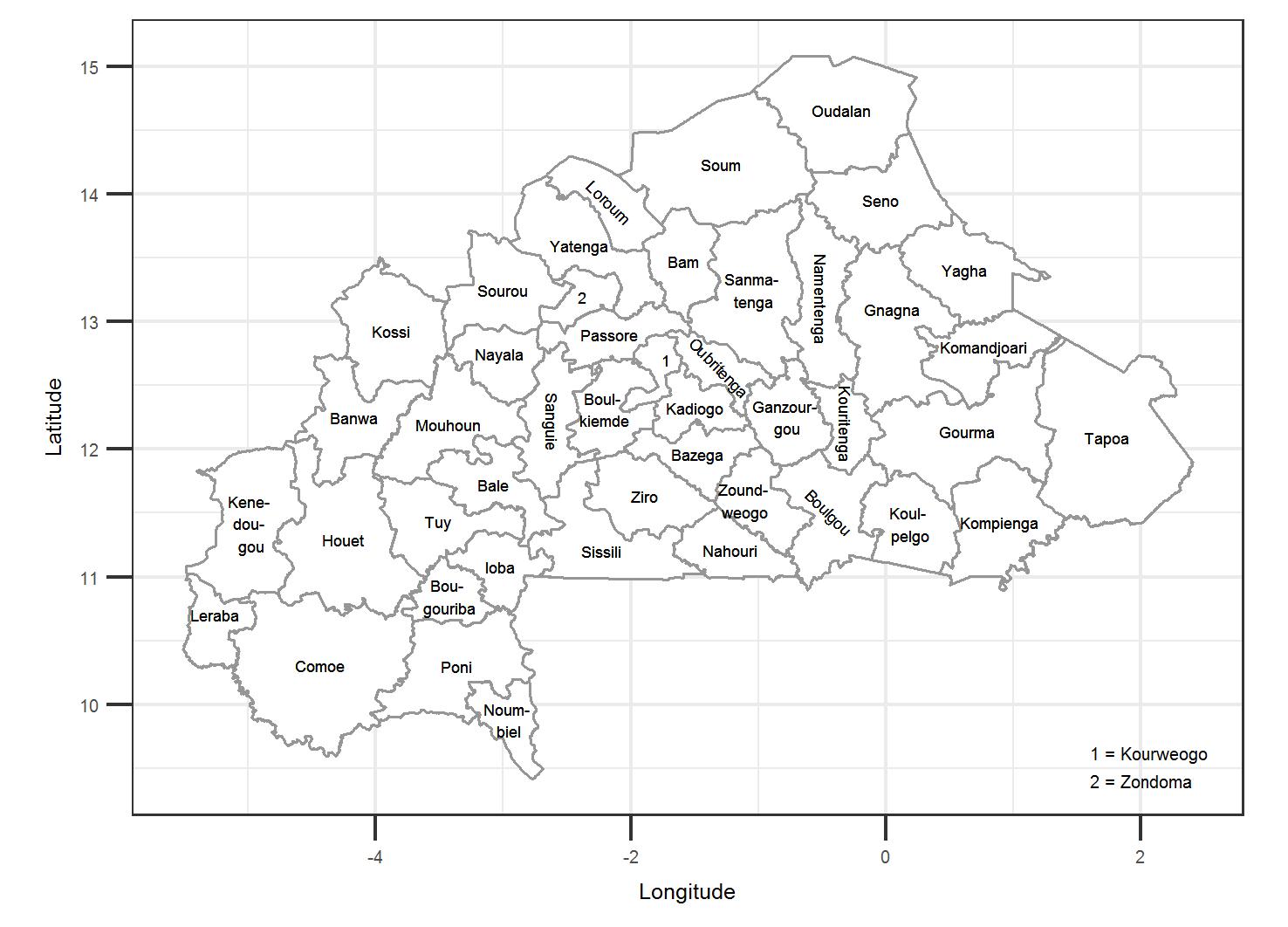


SI Fig. 2. Map of province names in Burkina Faso

# Performance of the forecast (out-of-sample variable selection)


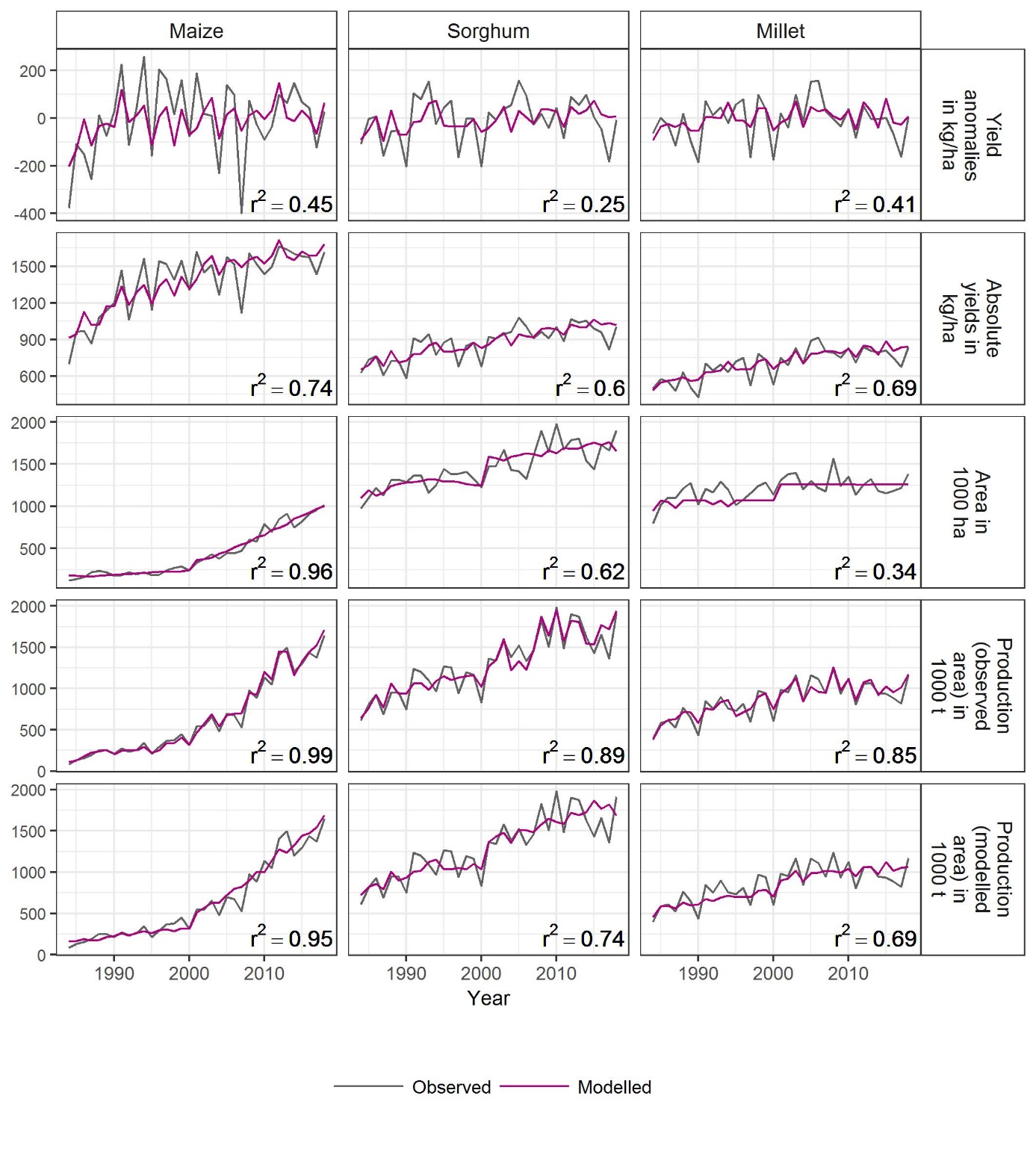


SI Fig. 3. Performance of the crop specific forecast with a lead time of one month for yield anomalies, absolute yields and harvest areas. The performance of the production forecast is shown for known harvest areas and for modelled harvest areas. The modelled yield data shows the result of the out-of-sample variable selection. The r^2^ values indicate the explained variance by each model. The crop specific forecasts were the basis for the aggregated forecast of all crops together. The lead time of the forecast for all crops is one month before the sorghum and millet harvest. Please note that at this point in time, maize is already harvested so that yields could be estimated based on weather influences of the whole growing season. For practical reasons, we chose the forecast for maize also with a lead time of one month to inform early on as soon as the forecast is available.

# Total population in Burkina Faso


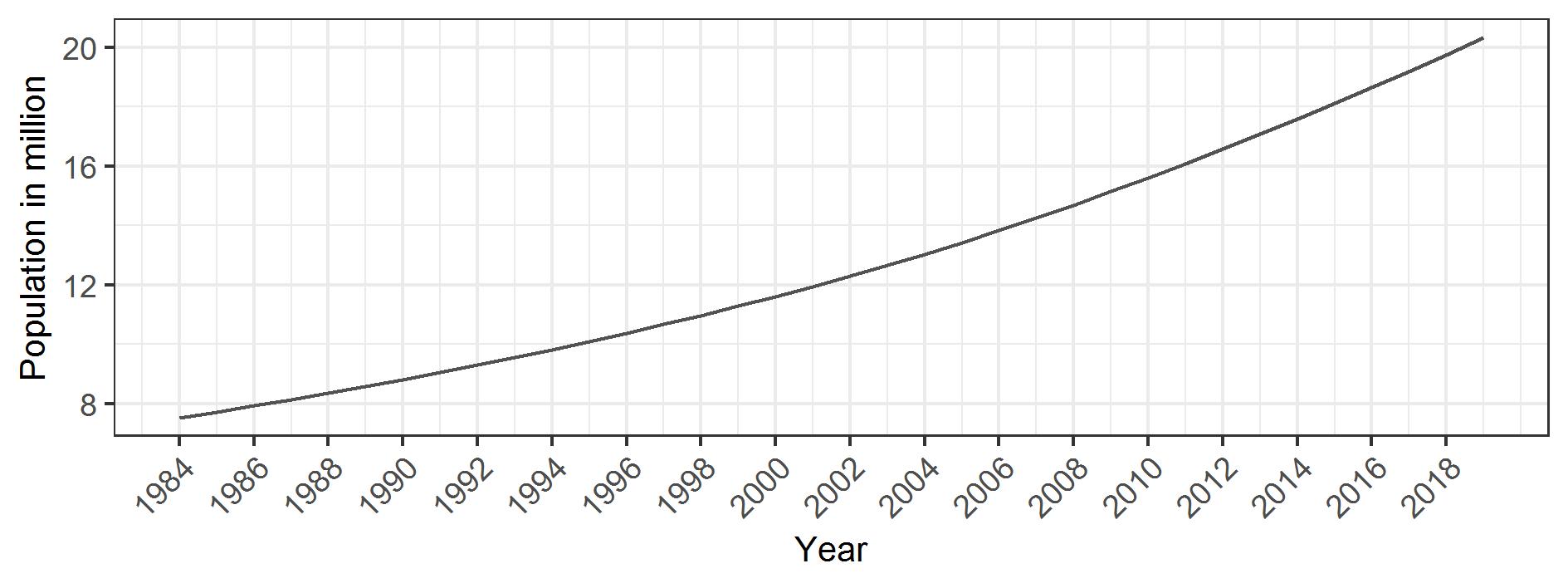


SI Fig. 4. Total population in Burkina Faso from 1984 to 2019, source: authors’ illustration based on World Bank (2020)^1^

# Trend in yield and harvest areas


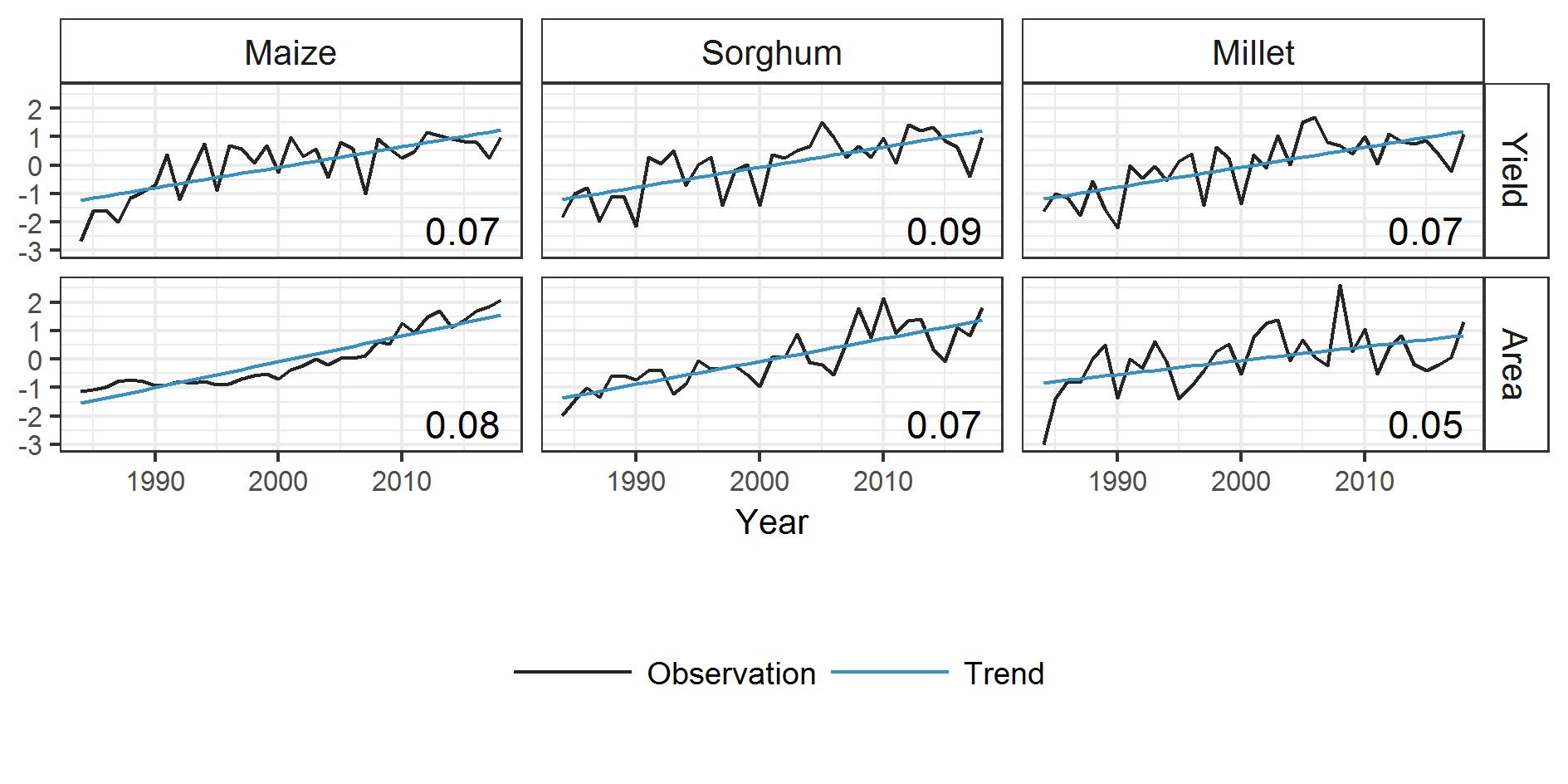


SI Fig. 5. Linear trend in yield and harvest areas for maize, sorghum and millet from 1984 to 2018; the y-axis shows standardised yields (upper panel) and harvest areas (lower panel). The slope of the trend is shown in the bottom right hand corner. A value of 0.07 means that there is a trend of 7% per year.

# Performance of the forecast (out-of-sample validation)


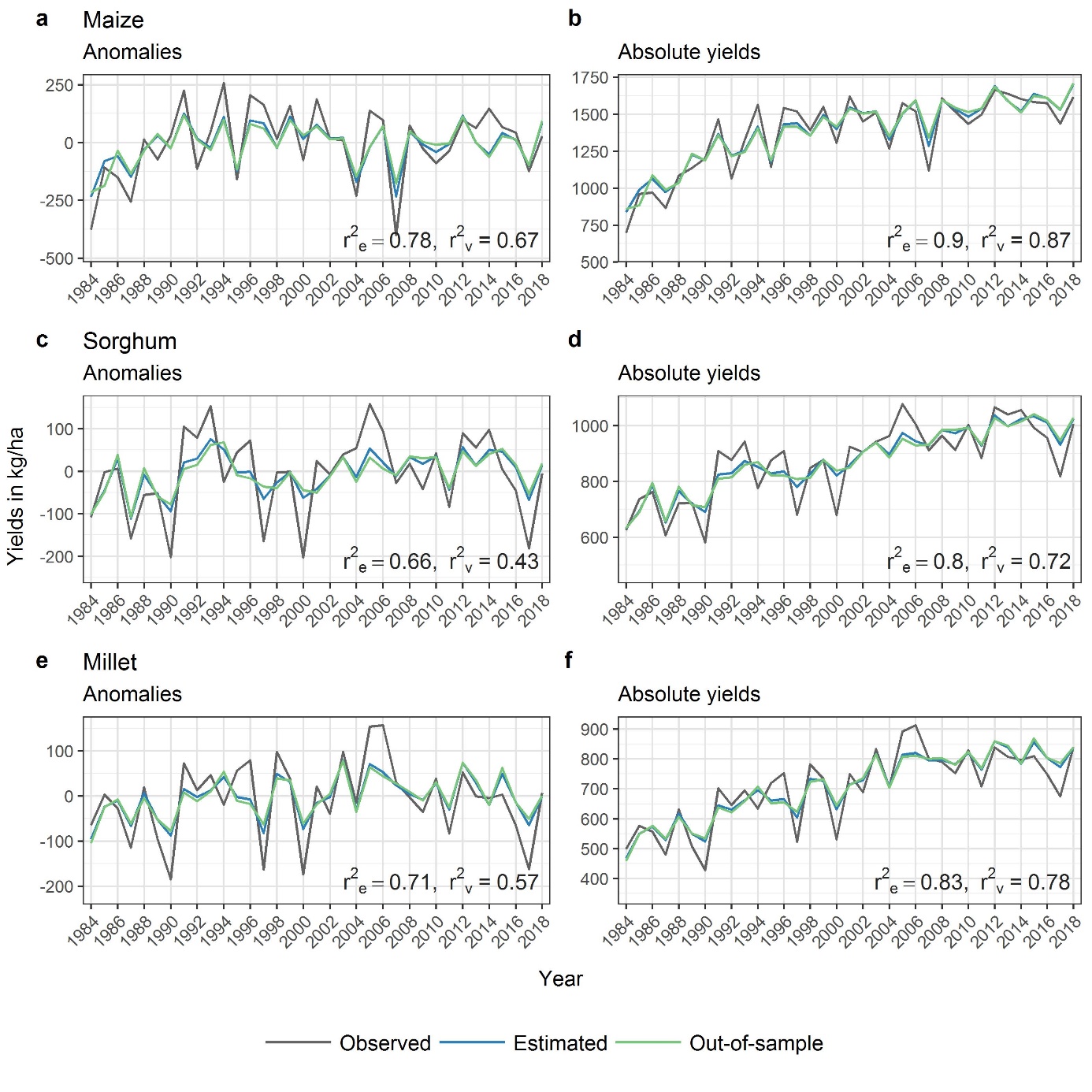
SI Fig. 6. Performance of the forecast with a lead time of one month for yield anomalies (left column) and absolute yields (right column) for maize, sorghum and millet from 1984 to 2018. The plot shows the observed yields in grey, the estimation results in blue and the out-of-sample validation results in green. The r^2^_e_ and r^2^_v_ values indicate the explained variance by each model, respectively.

# Performance of a simple production model


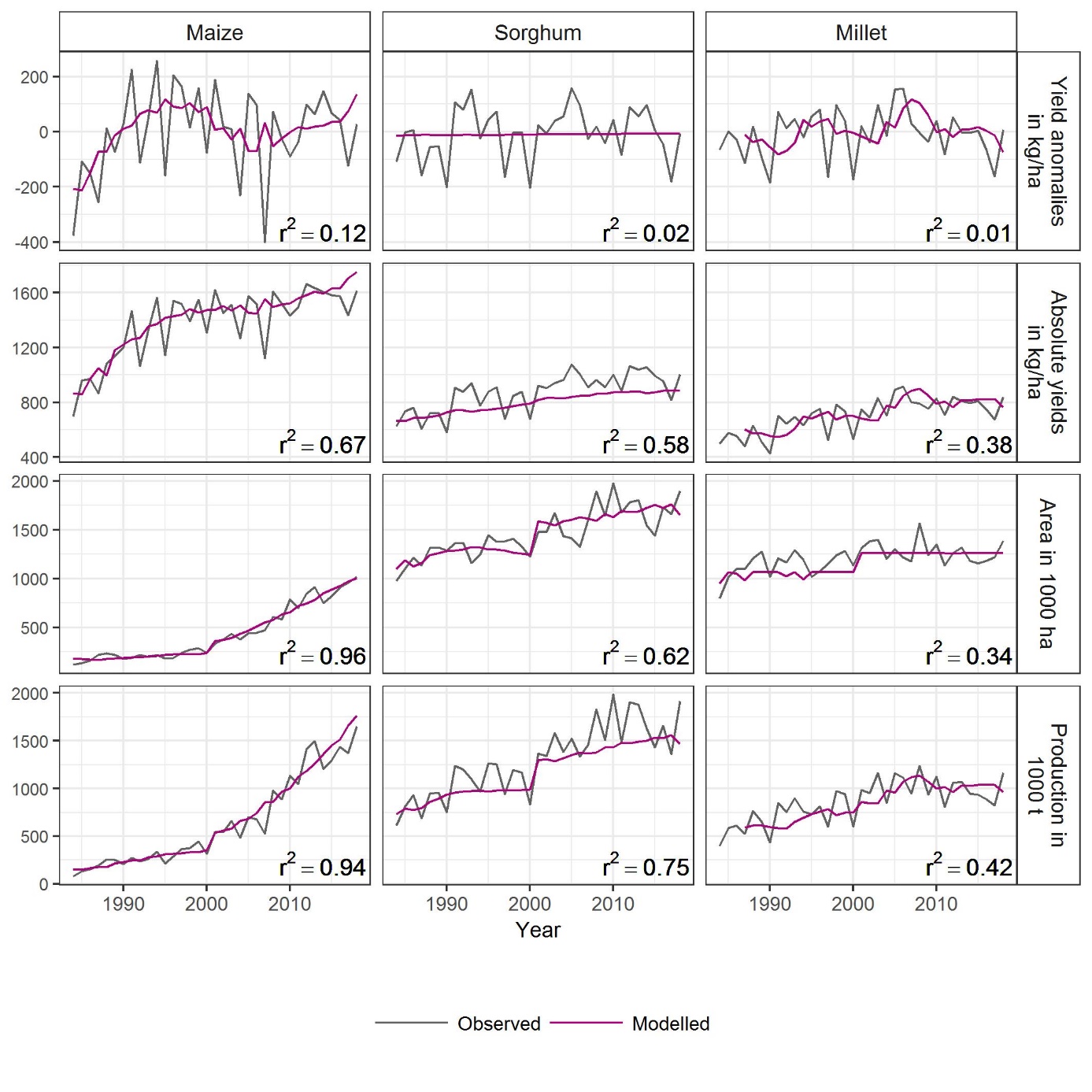


SI Fig. 7. Performance of a simple production model that is only based on yield and harvest area information from previous years. We tested the following four options: the median yield/harvest area, the median yield/harvest area of the previous three (five) years and the trend in yield/harvest areas calculated by a non-parametric LOESS function with a span of 0.9. For each crop, we chose the option that resulted in the highest correlation (Pearson’s r) between the observed and the modelled data. The best option for modelling yield is the trend calculated by LOESS for maize, the median of the last 3 years for millet and the median over all years for sorghum. The best option for modelling harvest areas is the trend calculated by LOESS for maize, the median over all years for millet and the trend calculated by LOESS for sorghum. This simple production model was set up to test whether a production forecast based on a weather-driven yield model is superior to a yield model based on yield information from previous years.

# Data cleaning of the annual production and harvest area statistics for maize, sorghum and millet on province level from 1984 to 2019

*SI Text 1.* We excluded observations with no harvest area or production as complete harvest losses are not likely on province level and are probably reporting errors. Yields were then calculated as production over harvest area. To guard against high outliers, yields outside the 95^th^ percentiles (2152 kg/ha for maize, 1571 kg/ha for sorghum and 1382 kg/ha for millet) were not considered. The mean value for the 0-95% percentile (95-100% percentile) is 1120 kg/ha (2937 kg/ha) for maize, 910 kg/ha (2641 kg/ha) for sorghum and 768 kg/ha (2538 kg/ha) for millet. Lastly, data for provinces with less than 10 years (i.e. one sorghum producing province and two millet producing provinces) were omitted to allow for robust model construction and validation by preventing overfitting. In sum, after data cleaning we used 1225 out of 1313 observations for maize, 1245 out of 1575 observations for sorghum and 1232 out of 1310 observations for millet. The statistics for sorghum showed unreasonable observations for the years 2012 and 2016 (i.e. no area and no production). Therefore we aggregated the times series for white and red sorghum which became available from 2003 on and used this data from 2003 on to guarantee a continuous time series for sorghum.

Even though some yield observations could not be used to validate the yield model results because of reliability issues as described above, they were still used in the national production aggregation to not skew aggregated production levels by omission.

# Crop specific lead time of the forecast


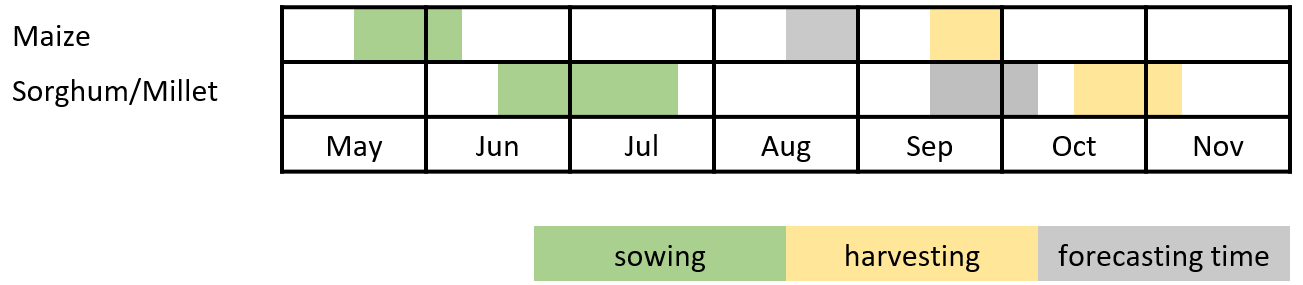


SI Fig. 8. Crop specific lead time of the forecast. The forecasting time is one month prior the harvest. The range in the forecasting time results from province specific sowing and harvesting dates^2^ (SI Fig. 13 and SI Fig. 14).

# Number of people affected by food insecurity and undernourishment in Burkina Faso


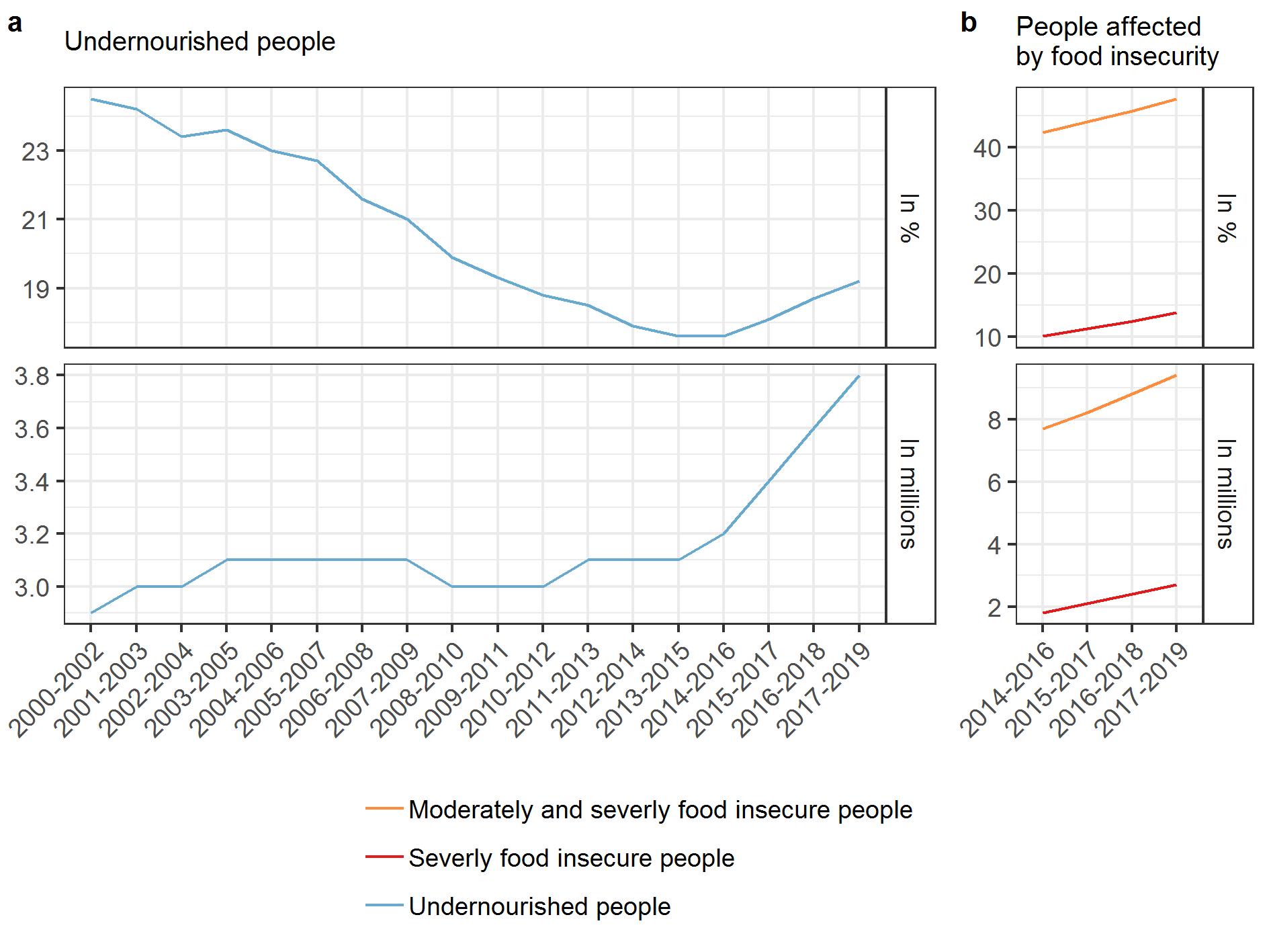


SI Fig. 9. People affected by food insecurity from 2000 to 2019 (plot a) and undernourished people from 2014 to 2019 (plot b) in Burkina Faso. The bottom panels show the absolute number of people, whereas the upper panels show the share of people in relation to the total population in Burkina Faso, source: authors’ illustration based on FAO (2020)^3^

# Consumed calories from maize, sorghum and millet compared to produced calories from these crops in Burkina Faso


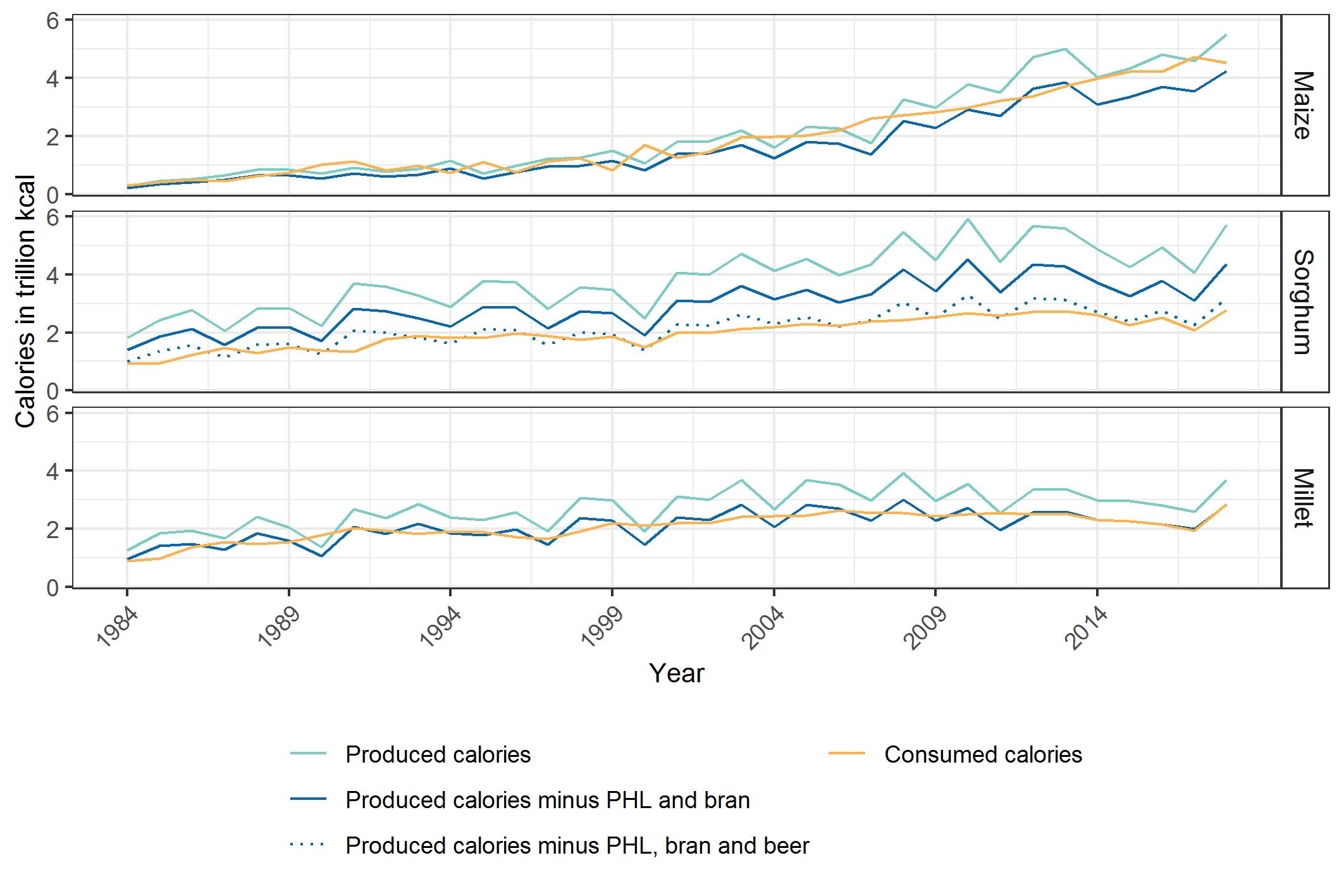


SI Fig. 10. Produced calories of maize, sorghum and millet compared to consumed calories from these crops on national level in Burkina Faso. Total produced calories^4^ are shown in light blue, whereas dark blue shows the produced calories minus post-harvest losses (PHL)^5^ and the bran^6^. Consumed calories were calculated by multiplying the supplied calories per person and day^7^ with the number of days per year and the total population in Burkina Faso^1^. Whereas the difference between produced and consumed calories from maize and millet can mostly be explained by PHL and the share of the bran in the crops (which is used for feed in Burkina Faso), a gap remains in the case of sorghum. FAO data suggests that on average 27% of total supplied calories from sorghum originated from sorghum beer in the time from 2014 to 2018^8^. The dotted line shows the produced calories from sorghum if in addition to PHL and the bran, average calories from sorghum beer were also subtracted. Despite the high agreement between this data and the consumed calories from sorghum, we did not include sorghum beer production in our analysis as this data is only available for five years and could not be extrapolated for the whole time period.

# Share of arable land in Burkina Faso


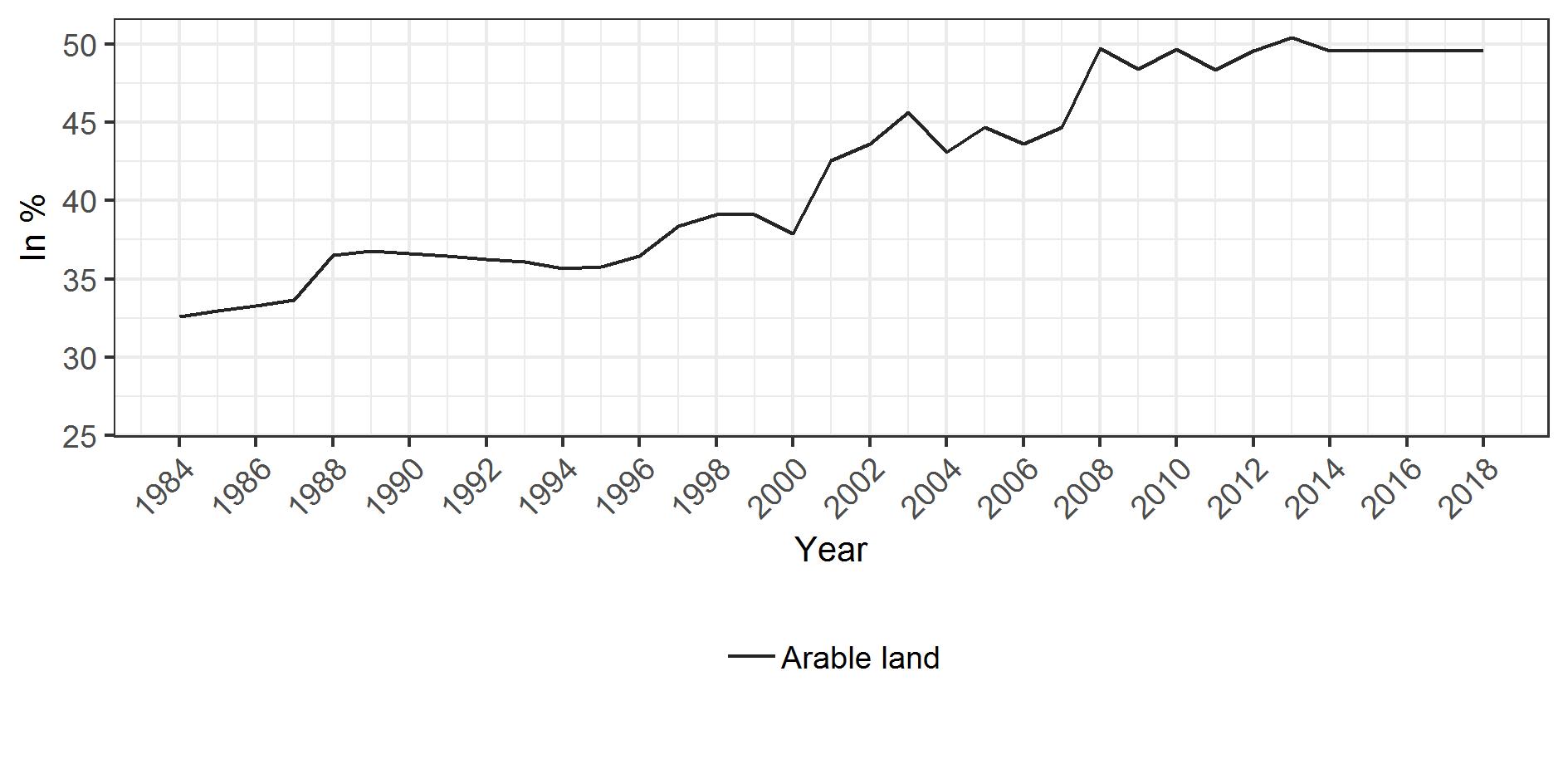


SI Fig. 11. Share of arable land in Burkina Faso from 1984 to 2018, source: authors’ illustration based on FAO (2020)^9^

# Software used in the analysis

*SI Text 2.* For our analysis, we used the statistical software *R -* version 4.0.5^10^ with the packages *tidyr*^11^ and *plyr*^12^ for data pre-processing, the packages *sp*^13^ and *rgdal*^14^ for spatial data processing, the package *glmnet*^15^ to perform LASSO regression and the package *ggplot2*^16^ to generate the figures and maps.

# Share of supplied calories from maize, sorghum and millet in the diet in Burkina Faso

**
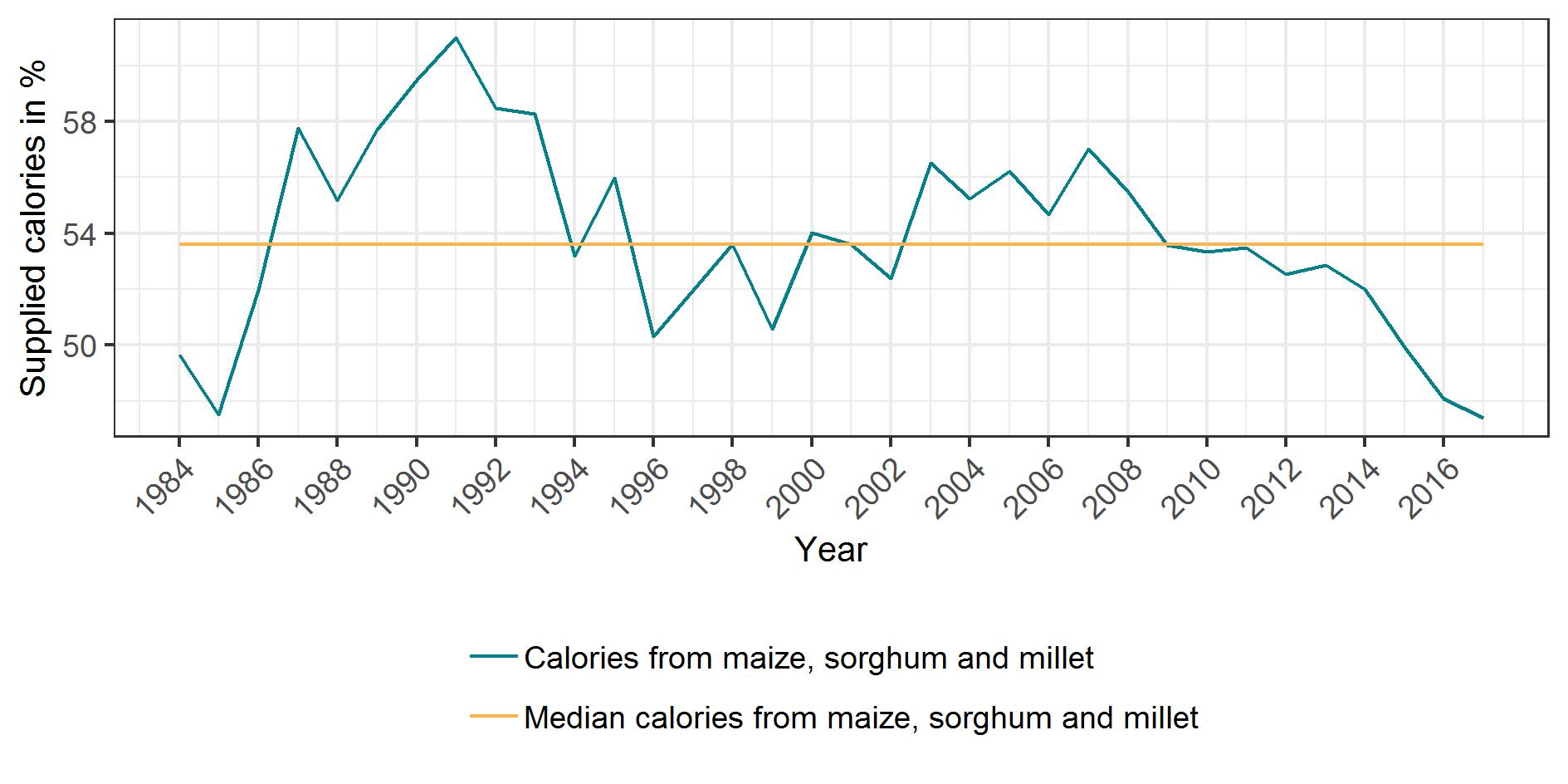
**

SI Fig. 12. Share of supplied calories from maize, sorghum and millet in total supplied calories per capita and day in Burkina Faso from 1984 to 2017; the yellow line shows the median value over the period 1984 to 2017, source: authors’ illustration based on (FAO 2020)^7^

# Equations for the calculation of vapour pressure deficit, growing degree days and percentile variables

**SI Eq. 1.** Calculation of vapour pressure deficit (VPD):

$$VPD=6.11*\exp\left( \frac{17.27*Tmax}{237.2+Tmax} \right)- \exp\left( \frac{17.27*Tmin}{237.2+Tmin} \right)$$

With *Tmax* as daily maximum temperature and *Tmin* as daily minimum temperature, formula according to Allen *et al* (1998)^17^.

**SI Eq. 2.** Calculation of growing degree days (GDD); *d* denotes the number of days within the growing season; *Days* denotes the total number of days within growing season:

$GDD=\sum_{d=1}^{Days} T_{d}^{GDD}T_{d}^{GDD}=\left\{ \begin{aligned} 0, &T_{d}<T^{Base} \\ T_{d}-T^{Base}, & T^{Base}\leq T_{d}\leq T^{Opt} \\ T^{Opt}-T^{Base}, & T_{d}>T^{Opt} \end{aligned} \right.$

With *T* as daily mean temperature; *T^Base^* as base temperature of 10°C; *T^Opt^* as optimal temperature of 30°C ^18^; *d* denotes the day within the growing season; *Days* denotes the total number of days within growing season.

**SI Eq. 3.** Calculation of percentile variables:

$var.p99=\sum_{d=1}^{Days} {var}_{d}^{var.p99}$ ${var}_{d}^{var.p99}=\left\{ \begin{aligned} 1, &{var}_{d}> p.99 \\ 0, &otherwise \end{aligned} \right.$

$var.p01=\sum_{d=1}^{Days} {var}_{d}^{var.p01}$ ${var}_{d}^{var.01}=\left\{ \begin{aligned} 1, &{var}_{d}< p.01 \\ 0, &otherwise \end{aligned} \right.$

With $var$ as the weather or sea surface temperature (SST) variable, and $p.99$ ($p.01$) as the 99% (1%) percentile of the weather or SST variable; the percentiles were calculated over all days of the vegetative and reproductive phase of the growing season within the time period of 2009 and 2018 for each region. As a sensitivity test, we also calculated the 5% (95%) and 10% (90%) percentiles, which provided similar results.

# Input variables for the yield model

| input name | Definition | unit |
| --- | --- | --- |
| Variables related to precipitation | | |
| psum | Precipitation sum | mm |
| pmedian | Median daily precipitation | mm |
| cdd5 | Consecutive dry days of equal or more than 5 days |  |
| cwd5 | Consecutive wet days of equal or more than 5 days |  |
| pB5 | Number of precipitation events below 5mm per day |  |
| pB15 | Number of precipitation events below 15mm per day |  |
| pA5 | Number of precipitation events equal or above 5mm per day |  |
| pA15 | Number of precipitation events equal or above 15mm per day |  |
| precip.p90 | Number of times the daily precipitation sum exceeds the 99% percentile of the daily precipitation sum |  |
| DWP | Number of days without precipitation |  |
| p.cv | Coefficient of variation of the daily precipitation sum |  |
|  |  |  |
| Variables related to temperature | | |
| tas.median | Median of the daily mean temperature | °C |
| tas.max | Median of the daily maximum temperature | °C |
| tas.min | Median of the daily minimum temperature | °C |
| tas.max.p95 | Number of times the daily maximum temperature exceeds the 95% percentile of the daily maximum temperature |  |
| tas.max.p05 | Number of times the daily maximum temperature falls below the 5% percentile of the daily maximum temperature |  |
| tas.min.p95 | Number of times the daily minimum temperature exceeds the 95% percentile of the daily minmum temperature |  |
| tas.min.p05 | Number of times the daily minimum temperature falls below the 5% percentile of the daily minimum temperature |  |
| tasmax.cv | Coefficient of variation of the daily maximum temperature |  |
| tasmin.cv | Coefficient of variation of the daily minimum temperature |  |
| Variables related to vapour pressure deficit | | |
| vpd.median | Median of the daily vapour pressure deficit | mm |
| vpd.p99 | Number of times the daily vapour pressure deficit exceeds the 99% percentile of the daily vapour pressure deficit |  |
| vpd.p01 | Number of times the daily vapour pressure deficit falls below the 1% percentile of the daily vapour pressure deficit |  |
| vpd.cv | Coefficient of variation of the daily vapour pressure deficit |  |

SI Table 2. Input variables for the yield model

*SI Text 3.* In addition to the median daily mean, maximum and minimum temperature over the growing season (*tas.median, tas.max, tas.min*), we included variables related to extreme temperatures. Temperatures above the optimum temperature range lead to a decline in the net photosynthesis rate because photosynthesis reduces with higher temperatures whereas respiration rates rise^19^. To account for extreme high temperatures, we included the number of days with a daily maximum temperature higher than the province-specific long term 99% percentile of the maximum temperature in the growing season (*tas.max.p99,* SI Eq. 3). Particularly low temperatures were represented by the number of times the daily minimum temperature fell below the province-specific long-term 1% percentile of the minimum temperature (*tas.min.p01*). Variations in maximum and minimum temperatures were represented by the coefficient of variation (*tasmax.cv* and *tasmin.cv*).

The overall water availability was represented by the precipitation sum (*Psum*) in the growing season. For optimal plant development, seasonal rainfall distribution and intensities are equally critical. Both excessive rain and drought stress can lead to crop failures and hinder timely planting and harvest^20^. To represent different precipitation ranges, we included the number of days with precipitation above a threshold of 5 and 15 mm (*pA5*, *pA15*, respectively) and below a threshold of 5 and 15 mm (*pB5, pB15, respectively*). We also included the number of days without precipitation (*DWP*), consecutive dry spells of more than five days (*cdd5)* and consecutive wet spells of more than five days *(cwd5)*. Extremely high precipitation events are covered by the number of times the daily precipitation sum exceeds the province-specific long-term 90% percentile of the daily precipitation sum. Variations in precipitation are covered by the median daily precipitation sum and the coefficient of variation of the precipitation sum.

Variables related to the vapour pressure deficit were included to account for water stress during plant growth. A high vapour pressure deficit leads to the closure of the stomata and therefore a reduction in carbon uptake from the atmosphere and thus crop yields^21^. As for temperature and precipitation, we included variables related to the median state (*vpd.median*), extreme low values (*vpd.p01*), high values (*vpd.p99*) and variations (*vpd.cv*).

# Crop calendar for maize, sorghum and millet


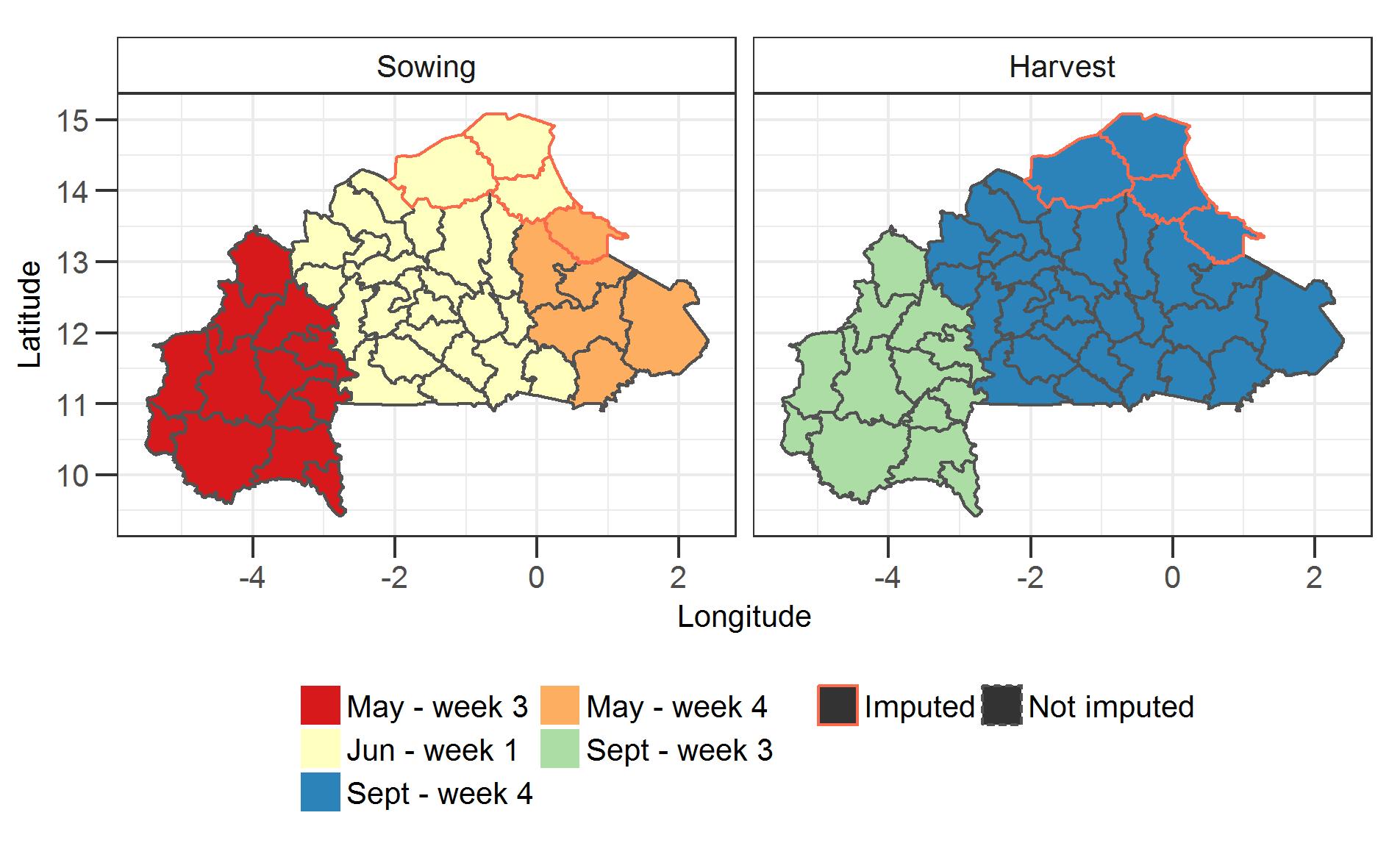


SI Fig. 13. Crop calendar for maize for Burkina Faso based on the FAO crop calendar^2^. Sowing is defined as the onset of the sowing period and harvest is defined as the end of the harvest period. The values represent the median onset of the growing season (left hand) and the median end of the growing season (left) over all available varieties (i.e. FBC 6, K.E.J. Barka, K.P.B. Wari, Espoir, SR 21, SR 22). Please note that information for the four Northern provinces Yagha, Soum, Seno and Oudalan in the Sahel zone (bordered in red lines) was not available. Therefore, we used the sowing and harvest dates of the neighbouring provinces.


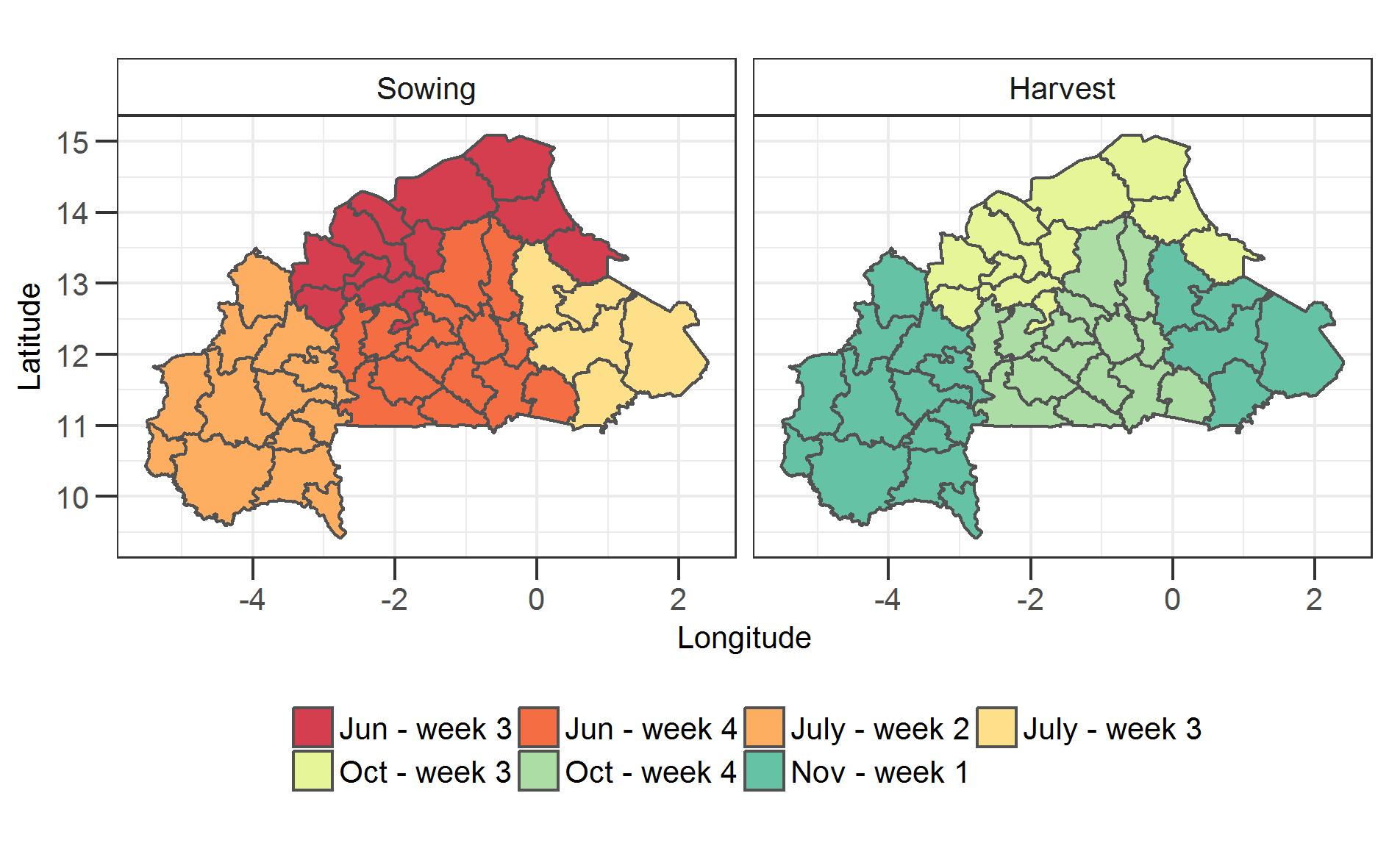


SI Fig. 14. Crop calendar for millet for Burkina Faso based on the FAO crop calendar^2^. Sowing is defined as the onset of the sowing period and harvest is defined as the end of the harvest period. The values represent the median onset of the growing season (left hand) and the median end of the growing season (left) over all available varieties of the crop calendar (i.e. IKMP1, IKMP2, IKMP3, IKMP5, IKMV 8201). This calendar was used for millet and for sorghum, because the FAO does not provide a separate calendar for sorghum and due to the similarity in sowing and harvest dates^22^

# Production, imports and exports of maize, millet and sorghum in Burkina Faso


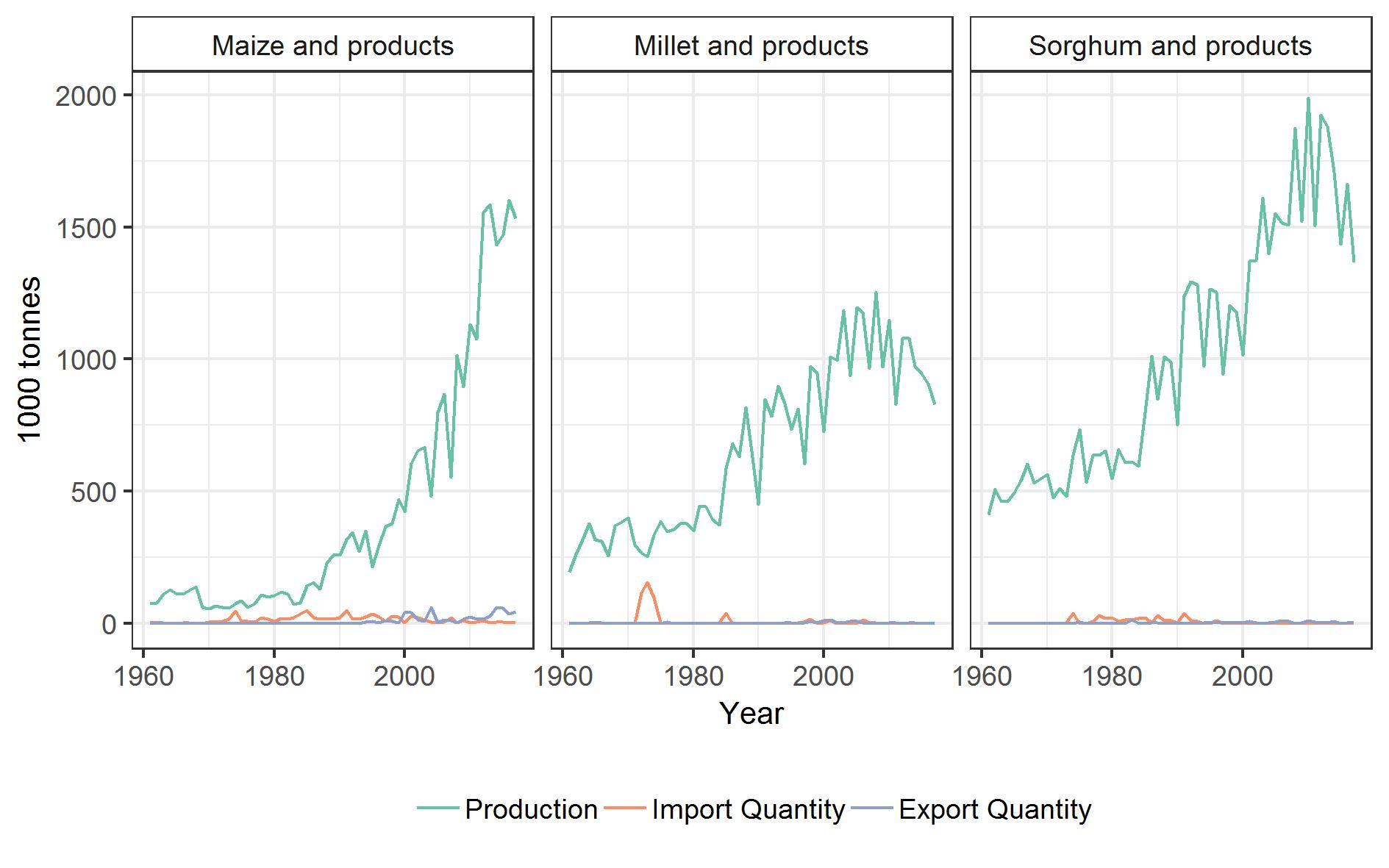


SI Fig. 15. Production, import quantity and export quantity of the crops maize, millet and sorghum in Burkina Faso from 1960 to 2017, source: authors’ illustration based on FAO (2020)^23^

# References

1. World Bank. Population, total. (2020). Available at: https://data.worldbank.org/indicator/SP.POP.TOTL?locations=BF. (Accessed: 3rd July 2020)

2. FAO. Crop calendar - An information tool for seed security. (2010). Available at: http://www.fao.org/agriculture/seed/cropcalendar/welcome.do;jsessionid=5FD03BC73666DEC13FB1C963D67932C9. (Accessed: 3rd July 2020)

3. FAO. FAOSTAT - Suite of Food Security Indicators. (2020). Available at: http://www.fao.org/faostat/en/#data/FS. (Accessed: 16th October 2020)

4. Ministère de l’Agriculture et des Aménagements Hydroagricoles / Direction Générale des Etudes et des Statistiques Sectorielles. *Données officielles de l’Enquête Permanente Agricole (EPA)*. (2020).

5. Aphlis. Dry weight loss: Burkina Faso - All crops - All years. (2020). Available at: https://www.aphlis.net/en/page/20/data-tables#/datatables?tab=dry_weight_losses&metric=prc&country=93&province=0. (Accessed: 3rd July 2020)

6. FAO. *Food composition tables for international use*. (1953).

7. FAO. FAOSTAT - Food Supply - Crops Primary Equivalent. (2020). Available at: http://www.fao.org/faostat/en/#data/CC.

8. FAO. FAOSTAT - Supply Utilization Accounts. (2021). Available at: http://www.fao.org/faostat/en/#data/SCL.

9. FAO. FAOSTAT - Land Use Indicators. (2020). Available at: http://www.fao.org/faostat/en/#data/EL. (Accessed: 16th October 2020)

10. R Core Team. R: A language and environment for statistical computing. (2018). Available at: http://www.r-project.org/.

11. Wickham, H. & Henry, L. tidyr: Easily Tidy Data with ‘spread()’ and ‘gather()’ Functions. (2019). Available at: https://cran.r-project.org/package=tidyr.

12. Wickham, H. The Split-Apply-Combine Strategy for Data Analysis. *Journal of Statistical Software* **40**, (2011).

13. Pebesma, E. J. & Bivand, R. S. Classes and methods for spatial data in R. (2005). Available at: https://cran.r-project.org/doc/Rnews/.

14. Bivand, R., Keitt, T. & Rowlingson, B. rgdal: Bindings for the ‘Geospatial’ Data Abstraction Library. R package version 1.3-2. (2018). Available at: https://cran.r-project.org/package=rgdal.

15. Friedman, J. *et al.* Regularization Paths for Generalized Linear Models via Coordinate Descent. *J Stat Softw* **33**, 1–22 (2010).

16. Wickham, H. ggplot2: elegant graphics for data analysis. (2009). Available at: https://ggplot2-book.org/.

17. Allen, R. G., Pereira, L. S., Raes, D. & Smith, M. *Crop evapotranspiration - Guidelines for computing crop water requirements - FAO Irrigation and drainage paper 56*. (1998).

18. Gilmore, E. C. & Rogers, J. S. Heat Units as a Method of Measuring Maturity in Corn. *Agron. J.* **50**, 611–315 (1958).

19. Barnabás, B., Jäger, K. & Fehér, A. The effect of drought and heat stress on reproductive processes in cereals. *Plant, Cell Environ.* **31**, 11–38 (2008).

20. Rötter, R. & Van De Geijn, S. C. Climate Change Effects on Plant Growth, Crop Yield and Livestock. *Clim. Change* **43**, 651–681 (1999).

21. Yuan, W. *et al.* Increased atmospheric vapor pressure deficit reduces global vegetation growth. *Sci. Adv.* **5**, 1–12 (2019).

22. FAO. GIEWS Country Brief Burkina Faso. *FAO* (2020). Available at: http://www.fao.org/giews/countrybrief/country/BFA/pdf_archive/BFA_Archive.pdf. (Accessed: 20th September 2020)

23. FAO. FAOSTAT - New Food Balances. (2020). Available at: http://www.fao.org/faostat/en/#data/FBS. (Accessed: 16th October 2020)
